# Quantitative LA-ICP-MS Imaging of Elemental Distributions with Protein Correlation in 3D Tumor Models

**DOI:** 10.1021/acs.analchem.6c03012

**Published:** 2026-07-30

**Authors:** Fatimah Zachariah Ali, Alexander P. Morrell, Piotr Robert Golda, Norfazlina Mohd Nawi, Premkamon Chaipanichkul, John M. McArthur, Pascal F. Durrenberger, Huda Alnufaei, Gary Royle, Kate Ricketts

**Affiliations:** † Division of Surgery and Interventional Science, Faculty of Medicine, University College London (UCL), 43-45 Foley St, London W1W 7TY, U.K.; ‡ Department of Biomedical Imaging, Faculty of Medicine, Universiti Malaya, South Tower, Universiti Malaya Medical Centre (UMMC), Jalan Universiti, Lembah Pantai 59100 Kuala Lumpur, Malaysia; § London Metallomics Facility, King’s College London (KCL), Franklin Wilkins Building, Stamford Street, London SE1 9NQ, U.K.; ∥ Institute for Biological Sciences, Faculty of Science, 37447 Universiti Malaya, Lembah Pantai, Wilayah Persekutuan, 50603 Kuala Lumpur, Malaysia; ⊥ Division of Radiation Oncology, Department of Radiology, Faculty of Medicine, 37690 Khon Kaen University, 123 Mittraphap Highway, Nai Mueang Sub-District, Mueang Khon Kaen District, Khon Kaen 40002, Thailand; # Department of Earth Sciences, UCL, 5 Gower Pl, London WC1E 6BS, U.K.; ∇ Respiratory Medicine, Faculty of Medicine, UCL, 5 University Street, London WC1E 6JF, U.K.; ○ Department of Medical Physics & Biomedical Engineering, UCL, Malet Place Engineering Building, Torrington Place, London WC1E 7JE, U.K.

## Abstract

Therapeutic efficacy in complex tissues depends on microscale drug and elemental distributions, yet quantitative mapping of these distributions in three-dimensional (3D) biological systems remains technically challenging. Laser ablation inductively coupled plasma mass spectrometry (LA-ICP-MS) enables sensitive elemental imaging, but quantitative application in heterogeneous 3D models is limited by preparation artifacts, calibration challenges and lack of validated integration with biological markers. Here we establish a validated workflow for quantitative LA-ICP-MS imaging in tumor spheroids, enabling spatial correlation with immunohistochemistry using consecutive sections. Systematic evaluation of preparation revealed substantial analyte redistribution under conventional conditions. Optimized cryo-embedding in 2% carboxymethyl cellulose combined with freeze-drying reduced peripheral boron leaching by ∼53% compared with gelatin-based media and ∼84% compared with OCT. Matrix-matched calibration (*r* ^2^ > 0.99) with endogenous ^31^P normalization enabled reproducible pixel-level quantification consistent with bulk ICP-MS measurements (*p* > 0.05). Consecutive section analysis showed protein expression varied by <20% between adjacent 30 μm sections, with radial profiles showing strong correlations across proteins and cell lines (Pearson *r* = 0.558–0.996), supporting spatial correlation of elemental and protein distributions. Applied to boron as a stringent low-mass analyte, the workflow achieved 10 μm spatial resolution with ∼7.4 ng g^–1^ detection limits. This approach provides a reproducible platform (Pearson *r* = 0.865–0.961, *n* = 3, *p* < 0.0001) for quantitative elemental imaging and cross modal spatial analysis in heterogeneous 3D biological systems.

## Graphical abstract

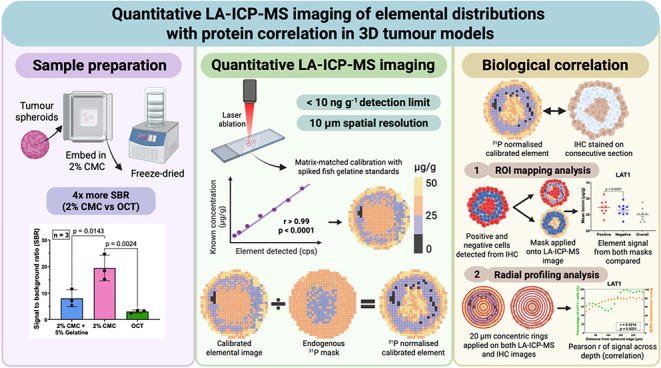

Quantitative spatial mapping of elements in biological systems remains a methodological challenge, particularly for achieving accurate and reproducible measurements at micrometre resolution in heterogeneous 3D samples. While many imaging approaches can visualize elemental distributions, quantitative determination is limited by preparation artifacts, matrix dependent signal variability, and the lack of validated analytical workflows.^1–7^ These limitations restrict mechanistic interpretation of elemental behavior in biological models and hinder comparison across studies.

Laser ablation inductively coupled plasma mass spectrometry (LA-ICP-MS) offers a powerful approach for spatially resolved elemental analysis, combining high sensitivity, broad elemental coverage, and micrometre-scale resolution. It has been widely applied to map metals, nanoparticles, and metal-based therapeutics in biological systems, including tumor spheroids, tissues, and brain models.^8–12^ Despite its sensitivity and multiplexing capability, applications in complex cellular models have largely remained qualitative or relatively quantitative.^13–15^ Quantitative implementation is hindered by methodological factors: sample preparation can redistribute elements, and matrix effects complicate signal interpretation, limiting reproducibility.

These challenges are amplified in 3D cellular samples, where structural heterogeneity is intrinsic. Regions with densely packed cells coexist with extracellular matrix–rich or necrotic zones, and such differences in cellular composition are expected to produce corresponding variation in LA-ICP-MS signals, which are reported as mass fractions (μg g^–1^). However, methodological variability introduced during cryo-sectioning can lead to irregular section thickness, resulting in pixels with higher cellular material being sampled purely due to cutting artifacts rather than biological differences. Because LA-ICP-MS signal intensity scales with the amount of ablated material, such thickness variations can bias apparent concentrations. Effective normalization is therefore required to correct for these section-to-section and pixel-level differences in sampled mass, ensuring that measured elemental variations reflect biological composition rather than technical variability. Although matrix-matched calibration approaches have been reported,^16–22^ a validated framework integrating sample preparation, calibration, endogenous normalization and correlative imaging for heterogeneous 3D systems remains lacking.

Boron was selected as the model analyte because of its clinical relevance to boron neutron capture therapy (BNCT), in which exogenously administered boron-containing agents such as boronophenylalanine (BPA) are selectively accumulated within tumors prior to neutron irradiation.^23^ Accurate quantification of intratumoral boron distributions is therefore of direct importance for BNCT dosimetry and treatment optimization. Beyond BNCT, boron-containing compounds are increasingly used in therapeutics, including the proteasome inhibitors bortezomib and ixazomib and benzoxaborole-based drugs such as crisaborole and tavaborole.^24^ From an analytical perspective, boron represents a particularly demanding test case because it is a low-mass element present at low concentrations^25–27^ and is highly susceptible to redistribution during sample preparation owing to its solubility and limited intracellular binding.^28–30^ Consequently, boron provides a stringent model system for evaluating the performance of quantitative elemental imaging workflows. This exogenous delivery format also has direct consequences for sample preparation. Unlike many endogenous trace elements, which are frequently protein-bound or compartmentalized — for example, iron sequestered within ferritin,^34^ zinc buffered by metallothionein,^35^ and copper distributed via dedicated metallochaperones^36^ — free boron is small, highly soluble, and lacks comparable intracellular binding partners. As such, it is expected to be unusually susceptible to diffusion and leaching during tissue preparation relative to elements retained through specific protein interactions. The boron-spiked spheroid model used here therefore represents a conservative, worst-case test of the sample preparation and quantification workflow. We hypothesize that elements with stronger biological retention may be less susceptible to redistribution than boron, although this was not directly evaluated here, and their direct assessment is an important direction for future work.

Here, we present a validated workflow for quantitative LA-ICP-MS imaging in heterogeneous 3D tumor spheroid models. We systematically evaluate fixation, embedding, sectioning, drying, calibration, and endogenous normalization to identify conditions that preserve spatial fidelity and analytical sensitivity. We show that endogenous phosphorus provides a robust internal normalizer, consistent with previous studies,^37–39^ and demonstrate that quantitative elemental maps can be integrated with biological context through correlation with immunohistochemistry on consecutive sections. Together, this work establishes an integrated and transferable framework providing a practical platform for quantitative elemental imaging and cross-modal spatial analysis in complex 3D biological systems, with applications spanning drug delivery, nanomedicine, toxicology, and metallomics.

## Experimental Section

### Cell Lines and Culture Conditions

Human colorectal cancer cell lines HT29 and HCT116 were obtained from the European Collection of Authorized Cell Cultures (ECACC, Sigma–Aldrich). Cells were maintained in DMEM (high glucose, GlutaMAX, Gibco) supplemented with 10% fetal bovine serum (Sigma–Aldrich) and 1% penicillin–streptomycin at 37 °C in a humidified incubator with 5% CO_2_ and atmospheric oxygen. Cells were routinely tested for mycoplasma contamination at 12-month intervals and were confirmed negative throughout the study. No additional short tandem repeat (STR) authentication was performed following acquisition.

Cumulative passage numbers during the study ranged from 7–18, and experiments were conducted using early passages (2–4) following thawing from frozen stocks. Cells were passaged at 70–80% confluence using standard trypsinisation.

### BPA Stock Preparation

BPA (17755, Sigma-Aldrich, U.K.) was dissolved in ultrapure water by adjusting the pH to ∼12 with sodium hydroxide. Fructose was added at a molar ratio of 1:1.5 (BPA:fructose), and the solution was vortexed until fully mixed. The pH was adjusted to 7.0–7.5 using hydrochloric acid to yield a final stock concentration of 20 mg mL^–1^. The solution was sterile-filtered (0.22 μm) and diluted in culture medium to a working concentration of 1 mg mL^–1^ for experimental use (50 μg B mL^–1^).

### Spheroids Formation and Treatment

Cells were seeded at 5 × 10^3^ cells per well in ultralow attachment 96-well plates (BIOFLOAT, Sarstedt, Germany) and cultured for 7 days to allow spheroid formation, with no media change. Spheroids were incubated with 2 μg mL^–1^ PI-containing BPA (1 mg mL^–1^) for 4 h prior to collection. For efflux experiments, spheroids were transferred to BPA-free medium and incubated for an additional 4 h. Each condition included 3 spheroids per replicate and was repeated in 3 independent biological experiments (9 spheroids in total). For each spheroid, a single central 30 μm section was selected for LA-ICP-MS analysis, with the immediately consecutive section used for IHC staining.

### Embedding Media Preparation and Sectioning Protocol

Following BPA treatment, spheroids were washed in ice-cold PBS and embedded in one of four media: optimal cutting temperature compound (OCT) (361603E, VWR), 5% gelatin (G/0150, Fisher Scientific), 2% carboxymethyl cellulose (CMC) (C4888, Sigma-Aldrich) or a gelatin–CMC mixture. Resin embedding was not evaluated as an alternative medium because standard resin protocols require chemical fixation and dehydration prior to resin infiltration, both of which were found to significantly alter boron spatial distribution and reduce measurable signal relative to fresh-frozen tissue (Figure 2). Since preservation of native boron localization was a primary objective of this study, embedding approaches requiring fixation were excluded from the study design.

For embedding, 5–7 spheroids were positioned within a Simport Base Mold 7 × 7 × 5 mm^3^ (HIS0220, Scientific Laboratory Supplies) and fully immersed in the respective medium. Samples were rapidly frozen by immersion in isopentane (24872, VWR) precooled on dry ice. Cryo-sectioning was performed in the dark (to preserve PI fluorescence) using a CryoStar NX70 cryostat (Epredia) with chamber and blade temperatures set to −20 °C (sample holder temperature varied by medium; see Supporting Table 1). Sections were cut at 30 μm thickness and mounted onto SuperFrost Plus slides (631–0446, VWR). Prior to embedding, brightfield images were acquired to record the diameter of each spheroid. To minimize variability arising from section depth, only sections with a measured cross-sectional diameter within 10% of the pre-embedding spheroid diameter were retained for analysis, ensuring that sections were taken at or near the equatorial plane where cross-sectional area is maximal. One section per spheroid was used for LA-ICP-MS analysis, with the immediately consecutive section used for IHC staining. The sections were immediately transferred to −80 °C storage, and freeze-dried for 2 h (Labconco Corporation) prior to LA-ICP-MS analysis to preserve spatial elemental distribution.

### Quantification Using B-Spiked Fish Gelatin

Quantitative boron imaging was enabled using matrix-matched calibration standards prepared by spiking fish gelatin with defined boron concentrations, implementing established gelatin-based calibration methodologies widely adopted for quantitative LA-ICP-MS bioimaging.^31–33^ Section below shows the calibration gels production in this project specifically.

#### Gelatin Solutions

A 10% (w/w) fish gelatin solution (Sigma-Aldrich, G7765) was prepared by dissolving 5 g gelatin in 50 mL ultrapure water (≥18.2 MΩ·cm, Milli-Q, Merck Millipore) with gentle heating and stirring until fully dissolved. A 1% (w/w) working solution was made by diluting 5 mL of the 10% stock into 45 mL of 1% nitric acid prepared from ultrapure water and trace-metal grade HNO_3_ (69% w/w; Fisher Scientific).

#### Multielement Calibration Standards

Matrix-matched calibration standards were produced by spiking the 1% fish gelatin solution with a custom multielement stock (1000 μg mL^–1^ each of Ag, B, Hf, Mo, Nb, Sb, Sn, Ta, Te, Ti, W, and Zr; High Purity Standards) to generate the concentration series detailed in Table 1. Gelatin droplet standards were prepared in house and analyzed using a total-ablation strategy to derive calibration coefficients, enabling conversion of elemental counts to absolute concentrations (μg g^–1^) for quantitative imaging.

**Table 1:** Preparation of Multi-Element and Gelatin Standards^a^

	concentration (μg mL^–1^)	multielement (mL)	1% HNO_3_(mL)
ME1	500	2.5	2.5
ME2	100	0.5	4.5
ME3	50	0.25	4.75
ME4	10	0.1	4.9
ME5	5	0.25	4.75
	concentration (μg mL^–1^)	stock solution used (mL)	1% fish gelatin (mL)
blank gel	0	0	10
gel 1	0.5	1 (ME5)	9
gel 2	1	1 (ME4)	9
gel 3	5	1 (ME3)	9
gel 4	10	1 (ME2)	9
gel 5	50	1 (ME1)	9

a Volumes of solutionsrequired to prepare 5 mL of multi-element standard (top) and 10 mL of gelatinstandards (bottom).

The relationship between measured counts per second and known boron concentrations found in this project was highly linear (*r* ^2^ > 0.99, Supporting Figure S2) and reproducible across three independent runs, supporting the use of this calibration strategy for absolute quantification within spheroid sections.

### LA-ICP-MS Instrumentation and Acquisition

Elemental imaging was performed using a 193 nm ArF* excimer laser ablation system (Iridia, Teledyne Photon Machines) equipped with a cobalt long-pulse ablation cell and coupled via an Aerosol Rapid Introduction System (ARIS) to a triple-quadrupole ICP-MS (iCAP MTX, Thermo Fisher Scientific). Instrument control and data acquisition were conducted using Qtegra software (Thermo Fisher Scientific).

Instrument performance was optimized using NIST SRM 612 glass to minimize laser-induced elemental fractionation (238U^+^/232Th^+^), maintain oxide formation below 1% (232Th16O^+^/232Th^+^), and maximize sensitivity of 59Co^+^, 115In^+^, and 238U^+^.

Spheroid sections mounted on glass slides were analyzed in fixed dosage raster mode with a 10 μm square laser spot, defining the spatial resolution. Imaging was performed with a laser energy density of 0.6 J cm^–2^, a repetition rate of 500 Hz (10 shots per pixel), and a helium carrier gas flow of 0.4 L min^–1^. Elemental detection was performed using Dynamic Reaction Cell (DRC) mode with oxygen as the reaction gas (0.16 mL min^–1^) to reduce polyatomic interferences on ^31^P and ^32^S. Two isotopes were monitored per acquisition (^11^B and ^31^P, or ^11^B and ^32^S), with dwell times of 14.3 ms (^11^B) and 1 ms (^31^P or ^32^S), a quadrupole total switching time of 4.7 ms, and a total acquisition time of 20 ms per pixel. A section thickness of 30 μm ensured that the imaging depth matched the physical section thickness. Slides were arranged within a four-position sample holder such that a boron-spiked fish gelatin calibration standard was analyzed within the same run to enable signal calibration.

### LA-ICP-MS Image Reconstruction

Elemental images were generated by combining the spatial coordinates from the laser-ablation system (Iridia, Teledyne Photon Machines) with time-resolved ion signals acquired by the ICP-MS (iCAP MTX, Thermo Fisher Scientific). Raw data streams were reconstructed into two-dimensional elemental maps using HDF-based Image Processing software (HDIP 1.9, Teledyne Photon Machines). Reconstructed data sets were exported and processed using a custom analysis pipeline implemented in Python (version 4). Negative intensity values arising from electronic noise were set to zero, and image-level masks were applied to remove peripheral artifacts and nonsample regions. For visualization, intensity values were scaled to the 95th percentile to enable comparability without altering quantitative outputs.

### Image Processing and Analysis

Boron signal (^11^B) was normalized to the phosphorus channel (^31^P), which served as an endogenous proxy for cellular mass, compensating for local variations in section thickness and ablated mass. Normalized boron concentrations were calculated according to eq 1

eq 1 *: Phosphorus-normalized boron concentration correction.*

1 B_corr=B_cal×(P_mean/P_pixel)

where; B_corr is the thickness-corrected boron concentration (μg g^–1^), B_cal is the matrix-calibrated boron concentration derived from the matrix-matched calibration curve (μg g^–1^), P_pixel is the raw ^31^P signal at each individual pixel (counts), and P_mean is the mean raw ^31^P signal across the entire section (counts). Pixels with near-zero phosphorus signal were excluded to avoid division artifacts.

The phosphorus map was additionally used to segment spheroid boundaries for quantitative analysis. Binary masks were generated by intensity thresholding the ^31^P signal followed by morphological filtering to remove isolated pixels and smooth edges. Only pixels within the mask were retained for quantitative analysis, excluding contributions from the surrounding embedding matrix.

Radial intensity profiles were calculated by normalizing pixel distances to the maximum spheroid radius derived from the segmentation mask. Mean normalized boron intensity (±SD) was calculated within angular bins and plotted as a function of normalized radial distance.

Signal-to-background ratios (SBR) were calculated by dividing the mean blank-corrected ^11^B intensity within the spheroid mask by the mean intensity in the surrounding nonmasked region of the same field of view. All processing steps were performed using custom reproducible scripts in MATLAB (2024b, MathWorks) to ensure consistent quantitative treatments across data sets.

### LOD and LOQ Calculations

To obtain the limit of detection (LOD) and limit of quantification (LOQ) of the LA-ICP-MS system, eqs 2 and[Disp-formula eq3] were used respectively, adapted from Lister.^37^

eq 2 *: Formula used to calculate the LOD of the LA-ICP-MS system.*

2 $$\mathrm{LOD}=\frac{\mathrm{gel}\left(\max\right)-\mathrm{gel}\left(\mathrm{blank}\right)}{g\mathrm{el intensity}\left(\mathrm{mean}\right)-g\mathrm{as blank}\left(\mathrm{mean}\right)}\times 3\times g\mathrm{as blank}\left(\mathrm{std}\right)$$

where; Gel (max) is concentration of highest gel (μg g^–1^); Gel (blank) is concentration of blank gel (μg g^–1^); Gel count (mean) is average counts of highest blank corrected gel (cts); Gas blank (mean) is average counts of gas blank (cts); and Gas blank (std) is standard deviation of gas blank (cts).

eq 3 *: Formula used to calculate LOQ of the LA-ICP-MS system.*

3 LOQ=3×LOD

where; LOD is the limit of detection calculated earlier (μg g^–1^).

### Volumetric Reconstruction of Spheroid Boron Content from LA-ICP-MS Sections

Three-dimensional estimates of total boron content were derived from two-dimensional LA-ICP-MS concentration maps. Pixel-resolved boron concentrations (μg g^–1^) were exported and analyzed in MATLAB (MathWorks). The geometric center of each spheroid section was estimated from the image centroid, and concentric annuli of equal radial width were generated. Mean boron concentration was calculated for each ring to obtain a radial concentration profile, *C*(*r*) (Supporting Figure S3).

Pixels outside the spheroid were excluded using a binary mask derived from the ^31^P channel to avoid radius overestimation. The maximal radial extent of the masked region was taken as the spheroid radius (*R*). Assuming spherical symmetry, the measured radial profile was extrapolated along the *z*-axis to approximate the full spheroid volume, with each concentric shell assigned its corresponding radial concentration.

The volume-weighted mean boron concentration (μg g^–1^) was calculated using eq 4.

eq 4 *: Formula used to calculate mean concentration of boron in spheroid section.*

4 $$C=\frac{\sum_{i}C\left(r_{i}\right)\times \left(r_{i+1}^{3}-r_{i}^{3}\right)}{R^{3}}$$

where *r_i_* and *r*_*i*+1_ denote the inner and outer radii of shell i.

Total spheroid volume was computed assuming spherical geometry (V = 4/3 πR^3^ *).* Spheroid mass was estimated by assuming a uniform density of 1 g cm^–3^ (1 × 10^–12^ g μm^–3^), and total boron content per spheroid was calculated (eq 5)

eq 5 *: Formula used to calculate the total boron mass per spheroid.*

5 $$m_{B}=C\times m_{\mathrm{spheroid}}$$

where *C* is the volume-weighted mean concentration (μg/g) and *m*_spheroid_ is the estimated spheroid mass (g). Yielding values are in ng per spheroid.

This approach enables direct comparison between spatially resolved LA-ICP-MS quantification and bilk ICP-MS measurements.

### ICP-MS Sample Preparation

For bulk quantification, spheroids were processed in parallel to LA-ICP-MS samples. Three spheroids per condition were collected into low-binding microcentrifuge tubes following incubation with BPA (4h, 37 °C). Samples were washed once with ice-cold PBS and pelleted by centrifugation (1500 rpm, 5 min). The supernatant was removed and spheroids were digested in 100 μL of 70% HNO_3_ (trace-metal grade, Sigma-Aldrich) at 60 °C overnight. Following complete digestion, samples were diluted in 2% HNO_3_ spiked with beryllium as an internal standard and analyzed using 7900 ICP-MS (Agilent). Boron concentrations were quantified against external calibration standards and expressed as total boron mass per spheroid.

### Proteins Correlation Analysis

To enable biological interpretation of elemental maps, LA-ICP-MS data were correlated with protein expression measured on immediately adjacent consecutive sections. PI staining was imaged prior to LA-ICP-MS analysis and used to identify nonviable regions within the section. Dead or necrotic cells can passively accumulate boron through loss of membrane integrity, independent of active transporter-mediated uptake; including such regions would therefore confound the correlative analysis between boron signal and protein expression in viable cell populations. Nonviable regions were accordingly excluded from analysis using a PI-derived mask together with spheroid boundary segmentation. To confirm that PI staining did not itself perturb the spatial distribution of boron, radial boron profiles were compared between PI-stained and unstained sections and found to be strongly correlated (Pearson *r* > 0.928, *p* < 0.0001; Supporting Figure S4).

To confirm that protein expression in IHC-stained sections was representative of the directly consecutive LA-ICP-MS section, protein expression was quantified across seven consecutive 10 μm sections from a single spheroid for LAT1, Ki67, and GLUT1. Expression varied by less than 20% across consecutive sections, supporting the validity of the consecutive-section coregistration approach used throughout this study (Supporting Figure S5).

Two complementary correlation approaches were applied:

1. Region-wise analysis quantified associations between masked, normalized boron intensity and corresponding IHC signal within aligned regions of interest (Supporting Figure S6a).
2. Radial profiling compared mean elemental and protein intensities as a function of distance from the spheroid center (Supporting Figure S6b).

### Flow Cytometry

Orthogonal validation was performed using flow cytometry to assess the BPA uptake in LAT1-defined subpopulations derived from HT29 and HCT116 dissociated spheroids. Spheroids were incubated with BPA (1 mg mL^–1^) and Hypoxia Green (1:2000, ThermoFisher Scientific) simultaneously in complete culture medium for 4 h at 37 °C. Fifteen spheroids per sample were collected and dissociated using Accutase (15 min, room temperature, ThermoFisher Scientific) with gentle pipetting every 5 min until a single-cell suspension was achieved. Cells were pelleted (1500 rpm, 5 min), resuspended in complete medium and stained with DAHMI (1:1000, Dojindo, Japan) and LAT1-AF647 antibody (1:1000, NBP2–50465AF647, BioTechne, U.K.) for 10 min at room temperature in the dark. Samples were analyzed immediately on a LSRFortessa X-20 flow cytometer (BD Biosciences), and at least 25,000 events were acquired per sample. Data were analyzed using standard gating to exclude debris and dead cells prior to quantification of BPA-associated fluorescence.

### Statistical Analysis

Statistical analyses were conducted using GraphPad Prism (v10.0, GraphPad Software Inc.). Data are presented as mean (±s.d.) unless otherwise indicated. Pairwise comparisons were performed using unpaired two-tailed Student’s *t* tests. Statistical significance was defined as *P* < 0.05. Correlations were evaluated using Pearson correlation coefficients; the degree of correlation was set based on criteria set by Akoglu et al. as shown in Supporting Table 3.^40^ Samples sizes (*n*) refer to independent biological replicates as indicated in the figure legends.

## Results

### Overview of the LA-ICP-MS Workflow

The overall experimental pipeline is summarized in Figure 1, outlining the sequential steps from spheroid preparation to quantitative image generation. The workflow integrates cryo-embedding, sectioning, freeze-drying, laser ablation, matrix-matched calibration, signal normalization, and correlative immunohistochemistry, providing a unified framework for spatially resolved elemental quantification in 3D tumor models.

**Figure 1:**
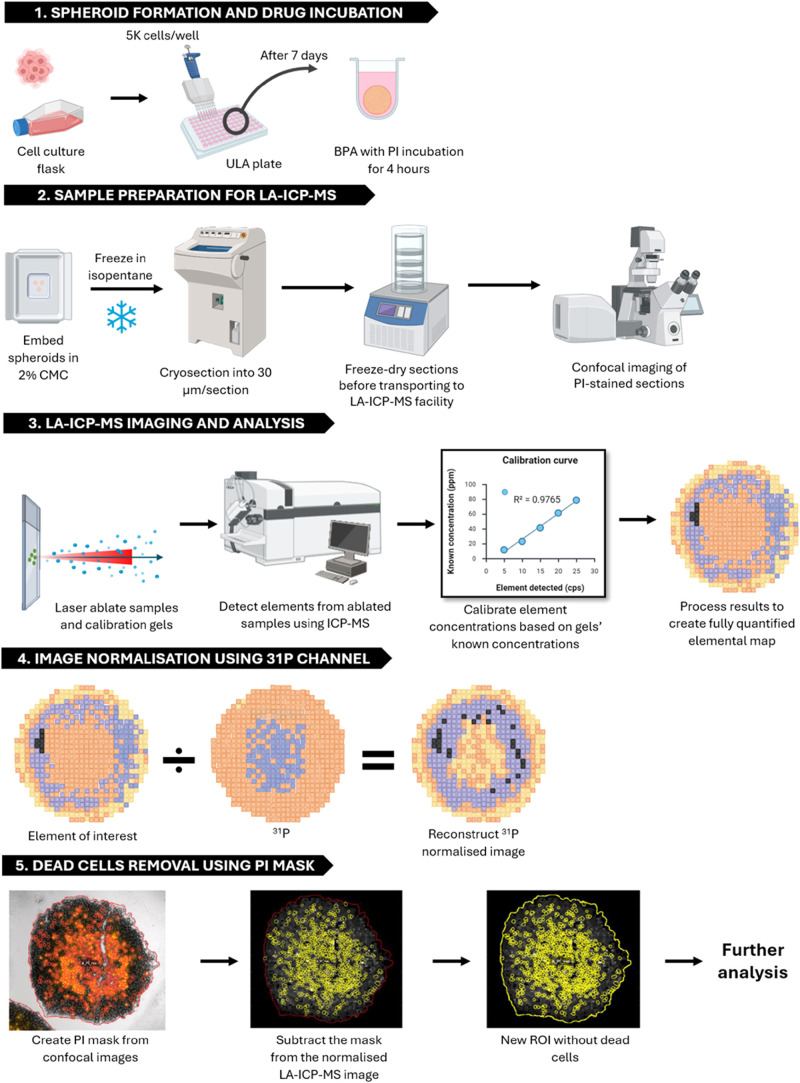
Integrated workflow for quantitative LA-ICP-MS imaging in tumor spheroids. (1) Tumour spheroids are generated from cultured cells and incubated with analyte. (2) Samples are cryo-embedded in 2% carboxymethyl cellulose, frozen, sectioned, and freeze-dried prior to imaging. (3) Sections and matrix-matched calibration standards are analyzed by LA-ICP-MS to generate quantitative elemental maps. (4) Elemental images are normalized using the endogenous ^*31*^P signal to correct for variations in ablation yield and tissue density. (5) Regions corresponding to nonviable cells are excluded using a propidium iodide (PI – in red) mask derived from confocal imaging, enabling downstream quantitative analysis.

### Optimisation of Sample Preparation for LA-ICP-MS Imaging

Reliable LA-ICP-MS imaging requires sample preparation conditions that preserves elemental localization and sample morphology while maintaining compatibility with downstream biological assays. We therefore systematically evaluated fixation, embedding, and drying procedures to establish conditions suitable for quantitative and correlative imaging.

#### Chemical Fixation Alters Spatial Distribution and Signal Intensity

Chemical fixation altered the boron distribution within tumor spheroids. Representative LA-ICP-MS images revealed differences in spatial signal patterns between fresh-frozen and 4% PFA-fixed samples (Figure 2a). To quantify these differences, radial intensity profiles were extracted from the spheroid center to the periphery. Fresh-frozen samples exhibited a steeper radial gradient, whereas PFA-fixed samples displayed a flatter profile, indicating reduced spatial contrast and altered apparent signal distribution (Figure 2b). In addition, fixation resulted in a significant reduction in mean normalized boron signal compared with fresh-frozen controls (Figure 2c). Together, these findings indicate that chemical fixation affects both the measurable boron signal intensity and its apparent spatial distribution under the conditions examined.

#### Embedding Medium Influences Spatial Confinement of Signal

Optimal cutting temperature media (OCT) and gelatin-based matrices showed boron signal extending beyond the spheroid boundary, whereas 2% carboxymethyl cellulose (CMC) preserved well-defined borders and spatial confinement of the boron signal (Figure 2d).

Quantitative radial profiling confirmed that boron leaching was present across all embedding conditions but was substantially reduced in 2% CMC. In OCT-embedded samples, peripheral boron signal at the spheroid boundary reached an average of 85% of the maximum intraspheroid signal, with a secondary signal peak of 37% detected approximately 1000 μm from the spheroid center, consistent with diffusion to neighboring spheroids. In 5% gelatin +2% CMC, peripheral signal was reduced to approximately 30% of the maximum. The lowest leaching was observed in 2% CMC alone, where peripheral signal was approximately 14% of the maximum intraspheroid signal, likely attributable to reduced diffusion pathways at the spheroid–medium interface during cryo-sectioning. (Figure 2e).

Signal-to-background ratios derived from phosphorus-based masks were at least 2-fold higher for CMC relative to alternative embedding media (Figure 2f), indicating superior spatial confinement of the analyte.

#### Freeze-Drying Preserves Signal Intensity and Spatial Fidelity

Freeze-dried sections from both HCT116 and HT29 spheroids exhibited at least 30% greater boron signal intensity and improved contrast relative to frozen-hydrated samples (Figure 2g).

Radial analysis showed sharper signal transitions at the spheroid boundary in freeze-dried sections, whereas frozen samples exhibited broader profiles and lower signal (Figure 2h), consistent with partial redistribution or loss of analyte during sample handling.

Quantitative comparison across both cell lines confirmed a significant increase in mean signal following freeze-drying relative to frozen (non–freeze-dried) controls, with 2.5-fold and 1.5-fold enhancements observed in HCT116 and HT29 spheroids, respectively (Figure 2i).

**Figure 2:**
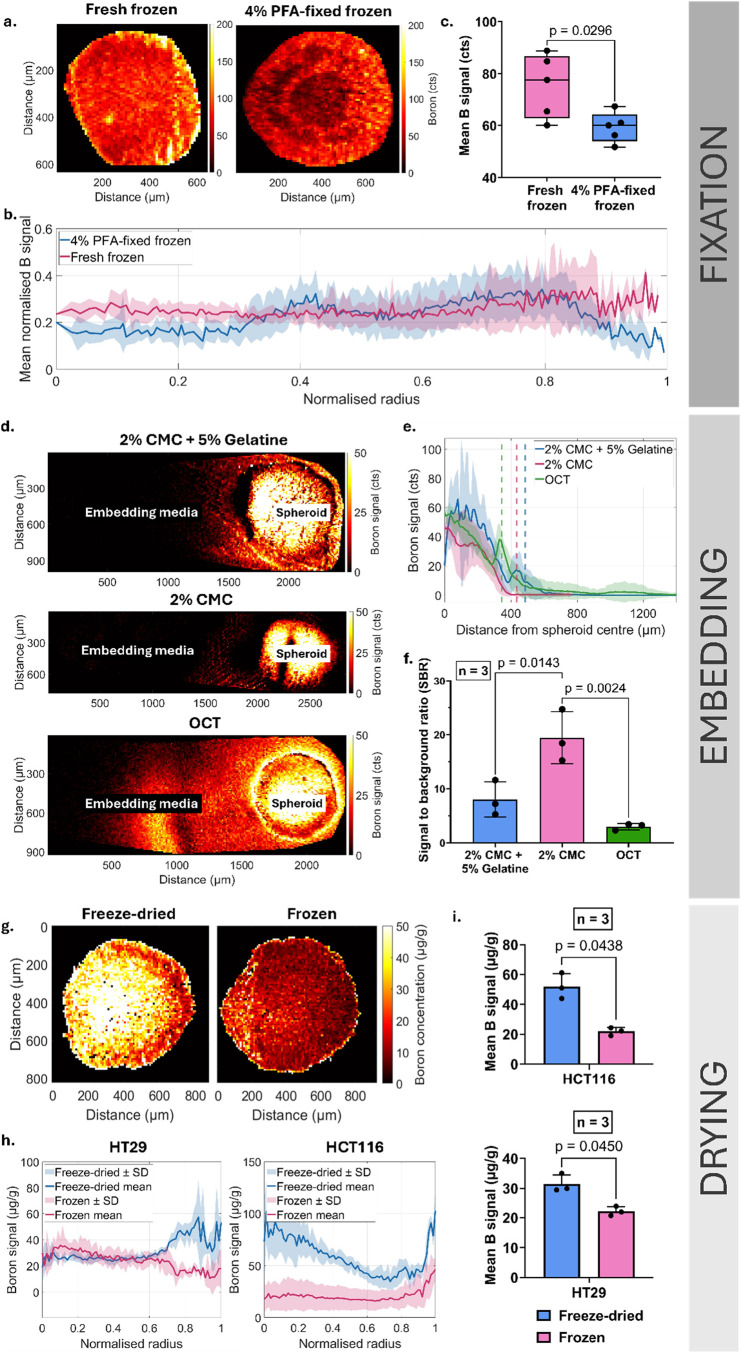
Optimisation of sample preparation for quantitative LA-ICP-MS imaging. (a) Representative boron maps from fresh-frozen and 4% PFA-fixed spheroid sections. (b) Radial boron intensity profiles with normalized distance (0 = center, 1 = edge) (mean ± s.d., *n* = 3 spheroids per group). (c) Mean normalized boron signal per spheroid under unfixed and fixed conditions (mean ± s.d., *n* = 5 per group; two-tailed *t* test). (d) Representative boron maps showing spatial distribution across embedding media. (e) Radial intensity profiles comparing embedding conditions from the center of spheroids through the embedding media (mean ± s.d). Dotted lines of the same color represent the edge of the spheroids (f) Signal-to-background ratios for each embedding medium (mean ± s.d., *n* = 3; two-tailed *t* test). (g) Representative LA-ICP-MS boron maps of unfixed HCT116 spheroid sections under frozen and freeze-dried conditions embedded in 2% CMC. (h) Radial intensity profiles with normalized distance (0 = center, 1 = edge) comparing drying conditions within unfixed HT29 and HCT116 spheroid sections, embedded in 2% CMC (mean ± s.d., *n* = 3). (i) Mean normalized boron signal across freeze-dried vs frozen spheroids from both cell lines (mean ± s.d., *n* = 3; two-tailed *t* test).

Based on these results, fresh-frozen, cryo-embedding in 2% CMC combined with freeze-drying was used for all subsequent experiments.

### Quantitative Performance of the LA-ICP-MS Workflow

#### Endogenous ^31^P Normalization Improves Morphological Fidelity in CMC-Embedded Sections

Raw boron maps acquired from CMC-embedded sections displayed spatial heterogeneity that did not correspond to underlying spheroid morphology, which may in part be attributed to variability in section thickness and ablation yield introduced during cryosectioning (Figure 3a,b). Endogenous normalization was therefore evaluated to correct for these preparation-induced intensity variations and improve morphological fidelity relative to the brightfield reference.

**Figure 3:**
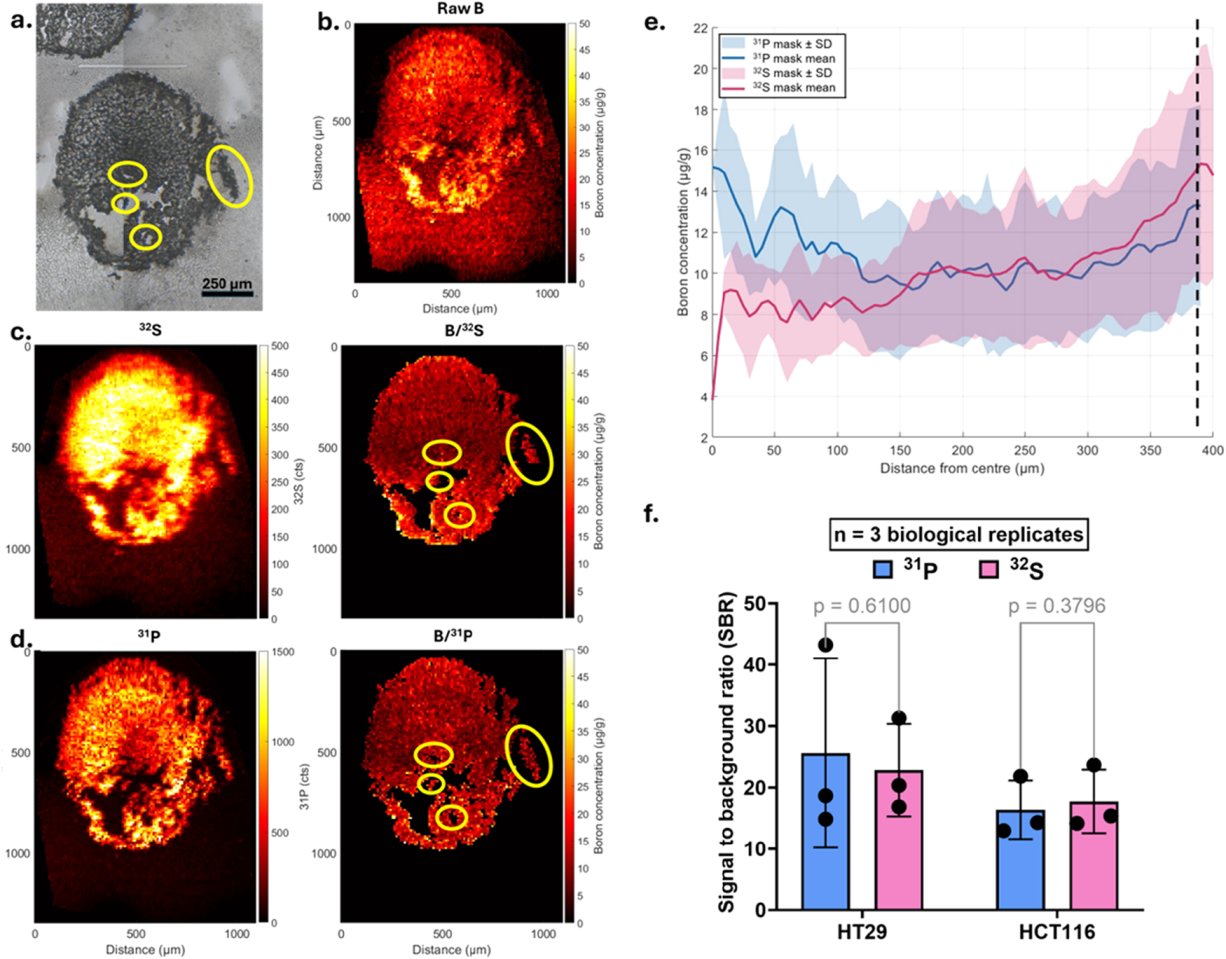
Evaluation of endogenous elements normalization for quantitative LA-ICP-MS of 3D spheroids. (a) Brightfield image of a representative HT29 spheroid section embedded in CMC media prior to ablation. Scale bar = 250 μm. (b) Raw boron map showing spatial heterogeneity across the section. (c) ^*32*^S signal image and boron distribution following normalization to ^*32*^S. (d) ^*31*^P signal image and boron distribution following normalization to ^*31*^P. Yellow circles indicate regions used to illustrate differences in morphology detail between normalization approaches. (e) Radial intensity profiles of B/^*32*^S and B/^*31*^P signals from normalized spheroid center to periphery. Black dotted line represents the edge of the spheroid defined using the brightfield images of the respective normalized images. (f) Signal-to-background ratios for each normalization approach in 2% CMC media across HT29 and HCT116 spheroids sections (*n* = 3 biological replicates).

Two endogenous elements, ^32^S and ^31^P, were evaluated as candidate normalizers. Both elements exhibited similar signal-to-background ratio across HT29 and HCT116 spheroids (Figure 3f). However, normalization to ^32^S, though widely used in LA-ICP-MS biological imaging due to its abundance in amino acids and structural proteins,^38^ incompletely corrected spatial heterogeneity, with residual intensity variations that were inconsistent with brightfield-defined morphology (Figure 3c, e).

In contrast, normalization to ^31^P yielded boron maps with spatial distributions closely matching brightfield morphology, confirmed by alignment of the spheroid boundary with annotated regions of interest (Figure 3d, e). Radial profiles of the normalized sections confirmed a steeper, morphologically consistent signal transition at the brightfield image-defined spheroid edge in B/^31^P compared to B/^32^S (Figure 3e).

Based on these results, ^31^P was selected as the endogenous normalizer for all subsequent quantitative LA-ICP-MS analyses.

#### Reproducibility of the LA-ICP-MS Imaging Workflow

Reproducibility of the optimized workflow was evaluated across independent experimental runs (*n* = 3). Representative boron distribution maps showed consistent spatial patterns and signal intensities when identical preparation, acquisition and processing parameters were applied (Figure 4a). Radial profiling demonstrated strong agreement in mean normalized boron signal, with limited dispersion throughout the spheroid radius (Pearson *r* = 0.865–0.961, *p* < 0.0001), (Figure 4b).

**Figure 4:**
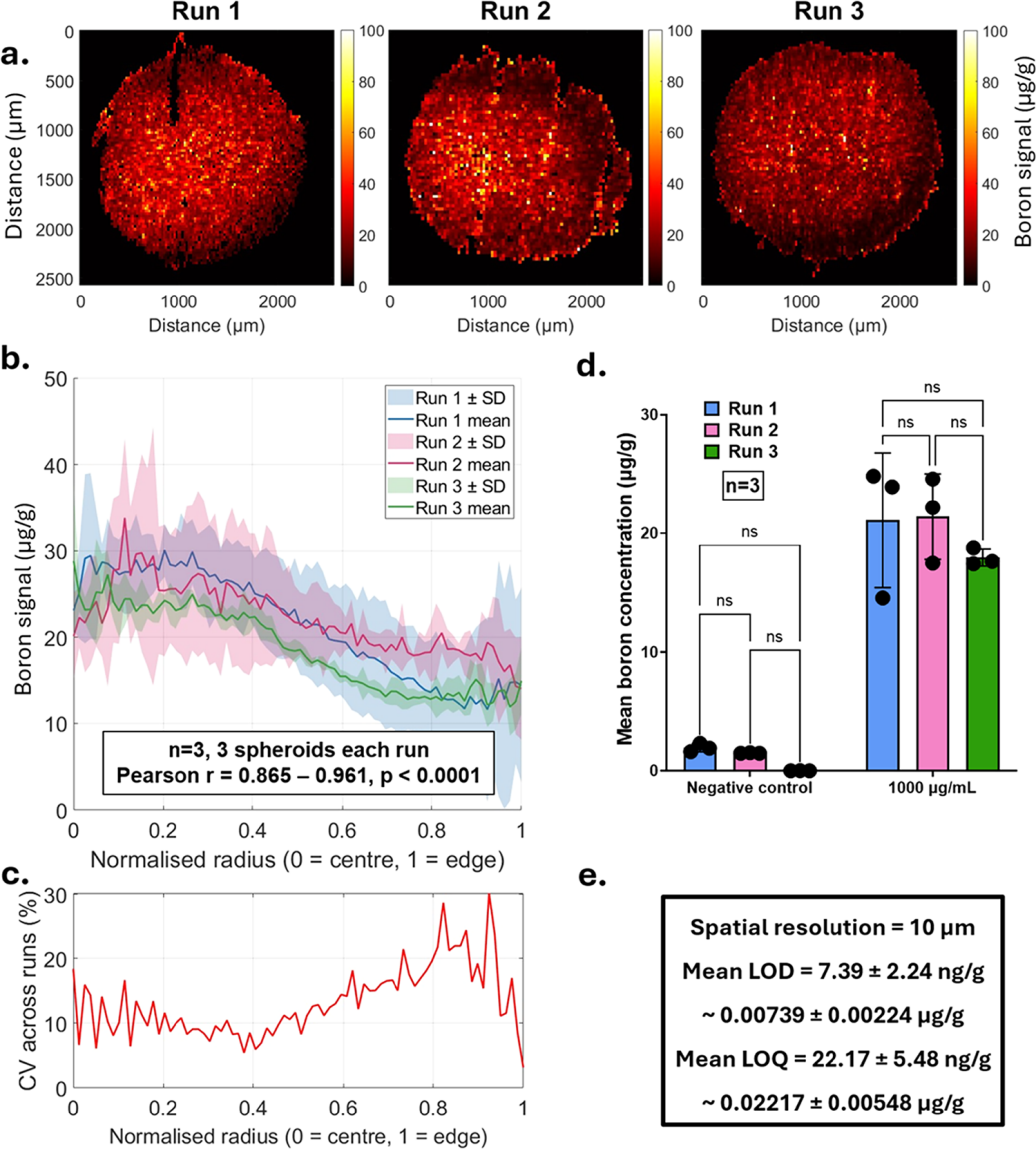
Reproducibility of the LA-ICP-MS imaging workflow. (a) Representative LA-ICP-MS boron maps acquired from three independent experimental runs using identical HT29 spheroid sample preparation and acquisition parameters; images are displayed using the same color scale. (b) Radial profiles of HT29 spheroid sections for three independent runs (mean ± s.d., *n* = 3 spheroids per run) (c) Coefficient of variation (CV) of boron signal across spheroid radius (*n* = 3, 3 sections per replicate). (d) Mean normalized boron signal per spheroid across three independent runs. Data are shown for HT29 spheroids incubated under different conditions: negative control and 1000 μg mL^–1^ BPA (mean ± s.d., *n* = 3 spheroids per run). (e) Summary of analytical performance metrics, including spatial resolution (laser spot diameter), limits of detection (LOD) and limits of quantification (LOQ) calculated across three independent runs.

The coefficient of variation (CV) remained below 20% across most of the spheroid (normalized radius 0–0.8), increasing modestly toward the periphery (0.8–1.0) but remaining below 30% (Figure 4c). This increase is consistent with reduced absolute signal intensity and greater sensitivity to boundary definition at the spheroid edge.

Mean normalized boron signal per spheroid did not differ significantly between runs (*p* > 0.05 across tested conditions, *n* = 3 spheroids per run; Figure 4d), indicating stable inter-run quantification.

A laser spot diameter of 10 μm defined the effective sampling resolution, comparable to cellular dimensions. Limits of detection (LOD) across three independent runs were 8.65, 4.81, and 8.71 ng g^–1^ (mean ± SD: 7.39 ± 2.24 ng g^–1^), confirming ng g^–1^-level sensitivity at micrometre-scale resolution (Figure 4e).

Together, these results demonstrate that the integrated workflow yields reproducible spatial and quantitative measurements across independent experiments.

### Integration of Biological Context into LA-ICP-MS Imaging

To enable spatial correlation between elemental imaging and protein expression, LA-ICP-MS maps were paired with immunohistochemical (IHC) staining acquired from immediately adjacent spheroid sections (±30 μm). Representative data sets illustrate the multimodal workflow, combining normalized boron distributions with brightfield imaging, propidium iodide (PI) staining, and IHC for GLUT1 and LAT1 (Figure 5a). PI staining was performed prior to freezing and cryosectioning to identify nonviable regions; all quantitative analyses were restricted to PI-negative regions to ensure correlation within viable cell populations.

**Figure 5:**
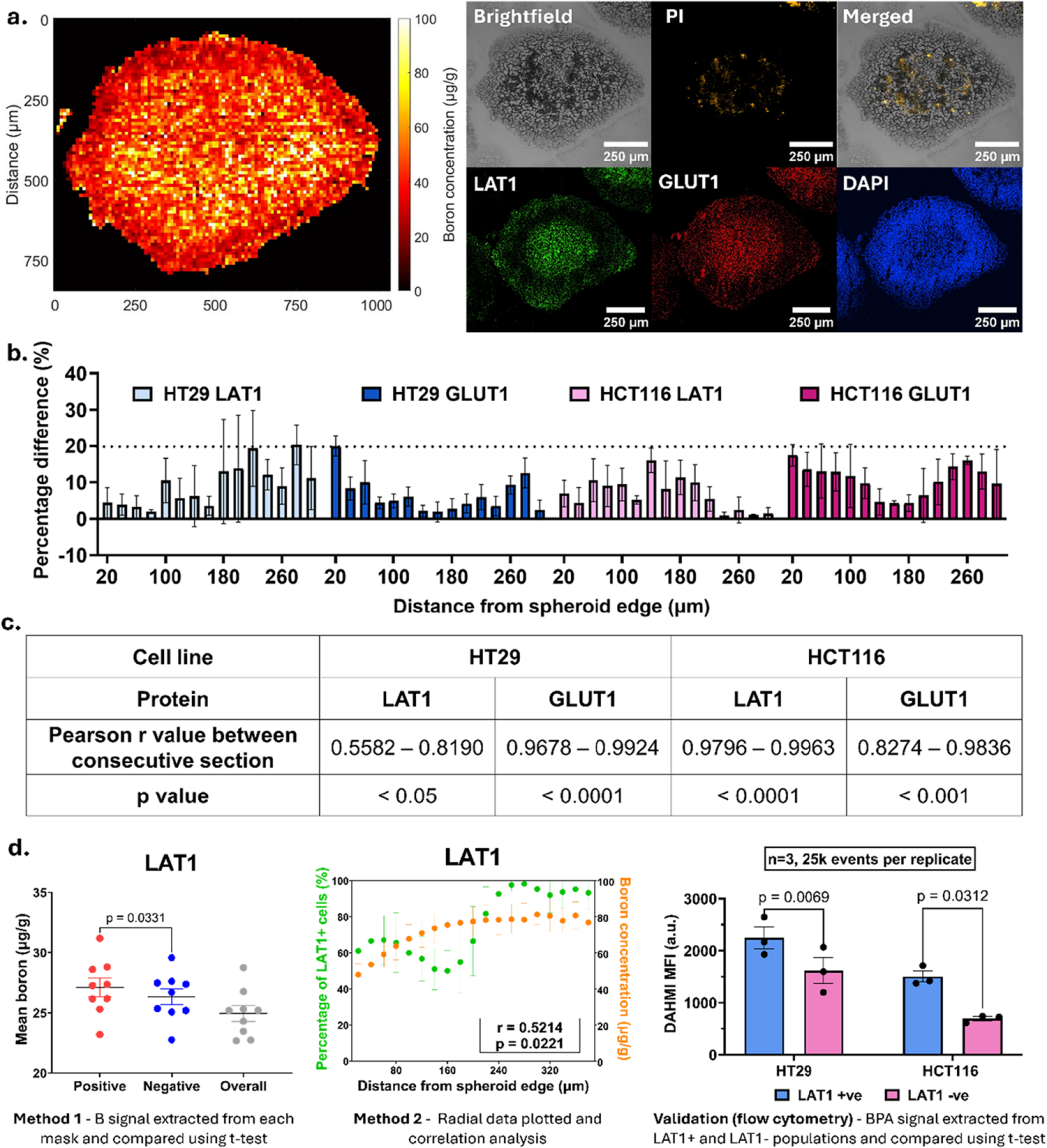
Correlative analysis of elemental distribution and protein expression in tumor spheroids. (a) Representative data sets used for correlation, including a normalized boron LA-ICP-MS map, brightfield image, propidium iodide (PI) staining, and immunohistochemistry (IHC) for GLUT1 and LAT1 from an immediately adjacent section. (b) Percentage variation in protein intensity between the LA-ICP-MS section and the immediately adjacent section (±30 μm) across GLUT1 and LAT1 in HT29 and HCT116 spheroids. (c) Pearson correlation of adjacent sections (±30 μm) across GLUT1 and LAT1 in HT29 and HCT116 spheroids (*n* = 3). (d) Validation of finding from analysis two analysis methods (*n* = 3, 3 spheroids per replicate) and flow cytometric validation of BPA uptake in LAT1-positive and LAT1-negative cell populations (*n* = 3, no. of events = 25, 000 each replicate). Statistical significance was determined using an unpaired two-tailed *t* test; exact p-values are as indicated.

To assess the validity of consecutive-section correlation, protein expression was first quantified across matched sections separated by ± 30 μm. Across HT29 and HCT116 spheroids, mean variation in staining intensity between adjacent sections was 10.5 ± 6.7% and 9.5 ± 4.7% for LAT1, and 6.6 ± 0.9% and 11.4 ± 2.1% for GLUT1, respectively (Figure 5b). Overall variability across all spheroids ranged from 0.9–23.7% (*n* = 3 spheroids per cell line).

Coefficients of variation were 7.4 ± 2.3% and 6.8 ± 2.7% for LAT1, and 4.9 ± 1.3% and 8.3 ± 1.9% for GLUT1 in HT29 and HCT116 spheroids’ consecutive sections, respectively, indicating that spatial protein distributions were largely preserved between consecutive sections at this scale.

Consistent with this observation, Pearson correlation analysis demonstrated strong spatial agreement between adjacent sections for most proteins analyzed. GLUT1 expression showed high correlation in both HT29, (*r* = 0.9678–0.9924) and HCT116 spheroids (*r* = 0.8274–0.9836), while LAT1 expression was highly correlated in HCT116 (*r* = 0.9796–09963) but more variable in HT29 (*r* = 0.5582–0.8190) (Figure 5c). All correlations were statistically significant (*p* < 0.05). Section-to-section variability must be quantified before assuming that a consecutive section faithfully represents the biology of the elementally mapped section; where this condition is met, consecutive-section correlation provides a reliable means of relating elemental and protein distributions.

Having established this framework, the known relationship between LAT1 expression and boronophenylalanine (BPA) uptake was used as a biological test case. BPA is transported into cells via the large neutral amino acid transporter LAT1, providing a mechanistic link between protein expression and intracellular boron accumulation.

Region-of-interest (ROI) analysis demonstrated that LAT-1 enriched regions exhibited higher boron signal compared to LAT1-low regions in HCT116 spheroids (*p* = 0.0331, paired two-tailed *t* test; *n* = 3, 3 spheroids per replicate). Radial analysis further revealed a positive correlation between LAT1 expression and boron distribution (*r* = 0.5214, *p* = 0.0221, Figure 5d), supporting spatial coupling between transporter expression and uptake.

Orthogonal validation by flow cytometry confirmed that LAT1-positive cells exhibited significantly higher BPA levels than LAT1-negative cells in both cell lines (36% and 39% increase in HT29 and HCT116, respectively; *p* < 0.05, *n* = 3, 25,000 events per replicate).

Together, these results demonstrate that combined LA-ICP-MS and consecutive section IHC workflow enables biologically meaningful spatial correlation between elemental distributions and tested proteins expression in 3D tumor models.

### Quantitative Validation of LA-ICP-MS Boron Measurements

To evaluate quantitative accuracy, LA-ICP-MS-derived measurements were compared with independent bulk ICP-MS analysis. LA-ICP-MS estimates agreed with bulk ICP-MS within <15% for samples above 3 ng boron per spheroid, whereas larger relative deviations (up to ∼50%) were observed only near background conditions (negative control and efflux).

Geometrically reconstructed total boron content showed strong correlation with bulk ICP-MS across conditions (*r* ^2^ = 0.9965; Figure 6a, b) supporting the validity of the calibration and reconstruction approach for estimating total uptake from spatial data.

**Figure 6:**
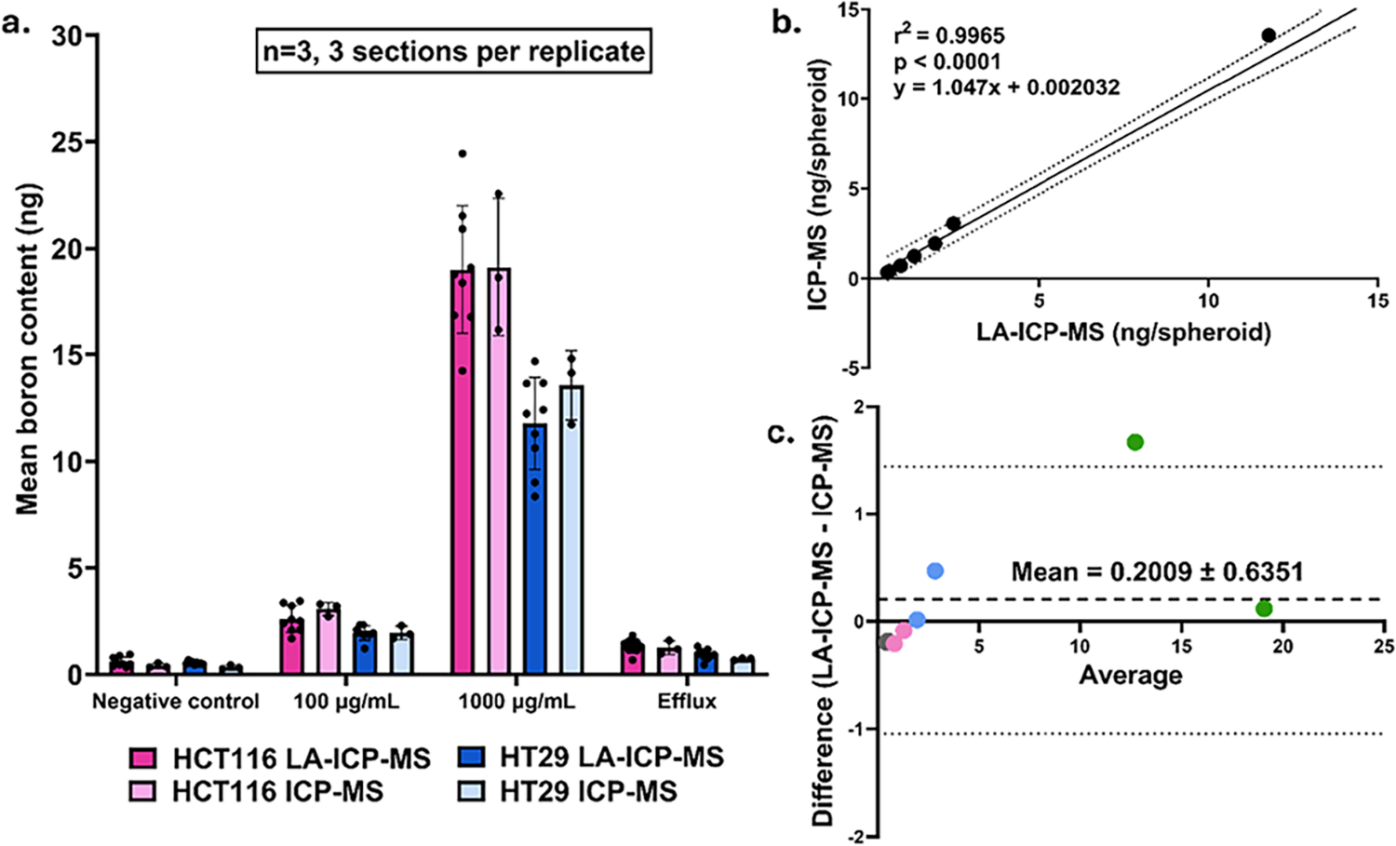
Volumetric reconstruction of spheroids from single LA-ICP-MS sections and validation against bulk ICP-MS. (a) Bar chart comparing B levels obtained from reconstructed spheroids (LA-ICP-MS) with bulk ICP-MS measurements. Data are shown for HT29 and HCT116 spheroids incubated under different conditions: negative control, 100 μg mL^–1^ BPA, 1000 μg mL^–1^ BPA, and 1000 μg mL^–1^ BPA + efflux (*n* = 3, 3 spheroids per replicate). (b) Total boron content per spheroid (ng/spheroid) estimated by volumetric reconstruction from LA-ICP-MS concentration maps assuming spherical geometry (SI > 0.9 confirmed for all spheroids; Supporting Figure S1). Values are reported in ng/spheroid to enable direct comparison with bulk ICP-MS measurements of total boron mass per spheroid. Pearson correlation between reconstructed LA-ICP-MS spheroid data and bulk ICP-MS measurements, showing a strong correlation (*r* ^2^ = 0.9965). Agreement demonstrates quantitative accuracy of the calibration and reconstruction approach. The dotted lines show the 95th percentile. (c) Bland-Altman plot assessing agreement between the two methods, with a mean difference of 0.2009 ± 0.6351. The green dots represent clinically relevant concentrations (>10 ng) while the gray, pink and blue dots represent the concentrations of ∼0.5 ng, ∼1 ng and ∼3 ng respectively.

Agreement between methods was further assessed using Bland–Altman analysis, which revealed a mean bias of 0.20 ng B with limits of agreement from −0.44 to 0.84 ng (Figure 6c). At therapeutically relevant exposure levels (1000 μg mL^–1^ BPA; > 10 ng B per spheroid), this bias corresponds to 1–2% of total uptake, well below typical biological variability. At intermediate uptake levels (100 μg mL^–1^ BPA; ∼3 ng B), the absolute deviation remained below ∼10% of measured uptake.

As expected, proportional variability increased under low-boron conditions (negative control, ∼0.5 ng B; efflux, ∼1 ng B), where measurements approach the lower limit of quantification and analytical noise contributes a larger fraction of the signal. Despite this, treatment-dependent differences between conditions on remained resolvable.

Together, these results demonstrate that the workflow provides quantitively consistent estimates of boron content across a biologically relevant dynamic range, with average percentage recovery found to be within 85–99% for concentration >3 ng, while maintaining micrometre-scale spatial resolution, supporting its application in mechanistic and comparative studies in 3D biological models.

## Discussion

In this study, we establish and analytically validate an integrated workflow for quantitative LA-ICP-MS imaging in three-dimensional biological systems, enabling pixel-resolved concentration mapping together with biologically interpretable spatial correlation to protein expression. This addresses a central limitation in the field, where quantitative elemental imaging in complex tissues remains challenging due to preparation artifacts, matrix effects, and the lack of integrated calibration and normalization strategies.

A primary determinant of quantitative accuracy was sample preparation. We show that commonly used embedding approaches, including OCT, introduce substantial artifacts including analyte redistribution into the surrounding embedding matrix. OCT embedding media are also known to cause ion suppression and spectral interference in mass spectrometry imaging.^39^ Our results demonstrate that cryo-embedding in 2% CMC combined with freeze-drying preserved both morphology and elemental localization while reducing analyte leaching by ∼84% relative to OCT. Previously, Buchholz et al. demonstrated that freeze-drying tissue sections followed by vacuum storage preserves analyte stability during shipping and storage prior to LA-ICP-MS imaging.^41^ These findings demonstrate that preparation protocols optimized for structural histology are not sufficient for elemental imaging, particularly for low-mass and diffusible analytes, and that preservation of elemental fidelity must be explicitly validated.

A second key advance is the systematic evaluation of matrix-matched calibration and endogenous normalization for quantitative LA-ICP-MS in heterogeneous 3D samples. While these approaches are widely used,^42,43^ they are rarely benchmarked in structurally complex systems where section thickness, cellular density and morphology vary spatially. Here, fish gelatin standards enabled linear calibration across the working range (*r* ^2^ > 0.99), supporting conversion of signal intensity to absolute concentration. Among candidate endogenous references, ^31^P provided more effective correction of section-induced variability than ^32^S, yielding improved morphological fidelity and sharper boundary definition in radial profiles, while maintaining comparable signal-to-background ratios across spheroids. These results demonstrate that the choice and validation of normalization strategy is a critical determinant of quantitative accuracy in heterogeneous samples, and that normalization strategies cannot be assumed to perform equivalently in heterogeneous 3D systems but must be empirically validated.

Under these optimized conditions, the workflow achieved 10 μm spatial resolution with sub-10 ng g^–1^ (0.01 μg g^–1^) detection limits, enabling measurement of multicellular concentration gradients at a single cell length scale. Reproducibility across independent runs was maintained below ∼20% CV across most of the spheroid, increasing modestly at the periphery due to reduced signal intensity. This level of reproducibility is consistent with reported limits for high-resolution LA-ICP-MS imaging, where ∼10–20% RSD (relative standard deviation, equivalent to CV) is reported at 5–15 μm spatial resolution in powdered samples or geological reference materials.^3,44^ Unlike those homogeneous samples, 3D tumor spheroids introduce additional variability from biological heterogeneity, including differences in cell density, batch-to-batch variation, and section boundaries. Quantitative accuracy was confirmed by comparison with bulk ICP-MS, with agreement within <15% for samples above 3 ng and strong overall correlation (*r* ^2^ ≈ 0.99). This places performance at the high end of previously reported quantitative elemental imaging approaches, which typically show 10–30% deviation.^45–47^ Larger relative deviations up to ∼50% were observed only near the lower limit of quantification, where absolute signal levels approach background noise. The three LOD values obtained across independent runs (8.65, 4.81, and 8.71 ng g^–1^; mean ± SD: 7.39 ± 2.24 ng g^–1^) reflect day-to-day variability in background signal and plasma stability, which can produce proportionally large percentage differences despite modest absolute differences at trace-level concentrations. Critically, all three LOD values fall in the single-digit ng/g range, far below the boron concentrations measured in untreated spheroid sections used as negative controls (approximately 1–3 μg g^–1^, equivalent to 1,000–3,000 ng g^–1^), and substantially below concentrations measured in BPA-treated spheroids (15–50 μg g^–1^). The day-to-day variation in LOD therefore has no practical impact on the quantitative conclusions of this study. These results indicate that the workflow provides accurate measurement of absolute concentrations, rather than relative signal distributions, across biologically relevant ranges.

The conventional LOD reported here is based on a fixed blank-signal threshold and does not account for local spatial contrast. Cernatič et al. recently proposed the Just Noticeable Difference (JND) as a complementary figure of merit for elemental mapping techniques including LA-ICP-MS, demonstrating that signals below the nominal LOD can remain visually discernible against a local background.^48^ Applying this framework to heterogeneous 3D data sets such as tumor spheroids represents a promising direction for future work.

Integration of elemental and biological data was enabled through correlation with IHC on adjacent sections. Protein expression varied by ∼10% on average between sections separated by 30 μm, with overall variation remaining within 0.9–23.7% depending on marker and cell line. Pearson correlations indicated strong spatial agreement for most proteins (e.g., GLUT1 up to *r* ≈ 0.99), although LAT1 in HT29 showed more modest correlations (*r* = 0.56–0.82), highlighting marker-dependent differences in section concordance. Variability between adjacent sections reflects multiple factors: true 3D biological heterogeneity (e.g., gradients in proliferation, hypoxia, necrosis) that changes cellular composition across depths, the relatively thick 30 μm sections that encompass multiple cell layers, technical differences in staining efficiency and antibody penetration, and small registration errors during alignment. Moreover, low absolute expression inherently yields larger relative variation for equivalent absolute differences, contributing to protein specific differences in percentage variation. This level of variability is consistent with reported values: serial section immunostaining studies typically report CV of ∼10–25% between adjacent slides depending on section thickness, marker and tissue.^49,50^ Importantly, the magnitude of section-to-section variability observed here remains substantially smaller than the biological gradients measured across spheroids (e.g., 2–5-fold differences between core and rim expression), indicating that technical variation does not dominate the spatial patterns analyzed. Nonetheless, because variability is marker dependent, validating section concordance for each protein remains essential when integrating serial sections for mechanistic interpretation of elemental and protein distributions in 3D systems.

Boron served as a stringent test analyte due to its low atomic mass, low endogenous abundance and high susceptibility to redistribution. Achieving ng g^–1^-level quantitative imaging at cellular length scales under these conditions, we hypothesize that the workflow is applicable to other low-abundance endogenous metals as well as therapeutic or toxicological elements, including platinum, gadolinium and iron-based agents. The framework is directly transferable to organoids and engineered tissues, where spatial heterogeneity presents similar challenges.

Limitations include the trade-off between spatial resolution and acquisition time^51^ inherent to laser ablation, and the assumption of radial symmetry used for volumetric reconstruction of spheroid boron content from single sections. Multicellular tumor spheroids are widely reported to develop radial gradients in proliferation, oxygen, and nutrient availability from the periphery to the core, and many experimental and mathematical models therefore treat spheroids as approximately radially symmetric systems to describe their growth and metabolic structure.^52^ To estimate the error introduced by the spherical approximation, we modeled the maximum volumetric deviation for ellipsoidal spheroids whose semiaxes vary by up to 10% from the mean radius, consistent with the observed SI > 0.9. Comparison of ellipsoidal volume (4/3 π abc) with the equivalent spherical volume (4/3 π r^3^) indicated a maximum error of approximately ± 10% in the most asymmetric cases, falling well below 5% for spheroids approaching true sphericity (Supporting Table 2). This level of uncertainty is considered acceptable given the inherent biological variability of multicellular spheroid models.

Volumetric reconstruction from two-dimensional (2D) section stacks is challenged by registration biases and deformation artifacts, which complicate coherent 3D volume generation.^53^ An important future extension of this workflow would be fully three-dimensional elemental reconstruction through sequential layer-by-layer ablation combined with profilometry-assisted registration, which could provide an alternative to physical sectioning for three-dimensional elemental mapping without the need for a simplified geometric approximation. Recent work has demonstrated the use of geometrical modeling and surface topography measurements to improve reconstruction accuracy in LA-ICP-MS imaging,^54^ and the application of such approaches to heterogeneous 3D biological systems such as tumor spheroids represents a promising direction for future methodological development. We note, however, that the heterogeneous cell density characteristic of multicellular tumor spheroids, with a quiescent or necrotic core surrounded by a proliferative outer rim, presents a practical challenge for uniform ablation depth across regions of differing density, which would require careful optimization of ablation conditions across the spheroid volume. In addition, broader interlaboratory benchmarking will be necessary to establish standardized performance metrics.

By integrating preparation optimization, calibration accuracy, endogenous normalization and cross-modal validation within a single framework, this work advances LA-ICP-MS from relative intensity imaging to a reproducible and quantitatively interpretable platform for elemental analysis in complex 3D biological systems.

## Conclusion

This study establishes a validated workflow for quantitative LA-ICP-MS imaging in heterogeneous three-dimensional spheroid models by systematically addressing key methodological determinants of accuracy, including sample preparation, calibration, endogenous normalization, and cross-modal validation. Optimized preparation (2% CMC cryo-embedding with freeze-drying) reduced analyte loss by ∼84% relative to OCT, while matrix-matched gelatin standards enabled linear calibration (*r* ^2^ > 0.99) and conversion of signal intensity to absolute concentration. Under these conditions, the workflow achieved 10 μm spatial resolution with ng g^–1^-level sensitivity (LOD 7.39 ± 2.24 ng g^–1^) and reproducible quantification across independent runs (CV < 20% across most of the spheroid radius).

Quantitative accuracy was confirmed by strong agreement with bulk ICP-MS (*r* ^2^ = 0.9965; < 15% deviation above ∼3 ng), demonstrating that reconstructed LA-ICP-MS measurements provide reliable estimates of total uptake. Integration with protein expression using consecutive-section analysis was feasible when section-to-section variability was characterized (typically ∼5–12% variation; CV < 10%), enabling biologically interpretable correlations between elemental distributions and molecular markers in multicellular systems.

While volumetric reconstruction requires simplifying assumptions (e.g., radial symmetry), these do not affect the spatial correlation analyses used to interpret elemental heterogeneity. By integrating preparation optimization, validated normalization, quantitative calibration and cross-modal benchmarking within a single framework, this work moves LA-ICP-MS from relative signal mapping toward reproducible, quantitatively interpretable imaging in complex 3D biological models, providing a practical platform for mechanistic studies of drug distribution and microenvironmental heterogeneity.

## Supplementary Material



## Data Availability

The data sets generated during the current study are stored in institutional data storage at University College London (UCL) and King’s College London (KCL). Data sets are available from the corresponding author upon reasonable request and subject to institutional data-sharing policies. Custom scripts used for LA-ICP-MS image processing, normalization, radial profiling and volumetric reconstruction were written in MATLAB (2024b, MathWorks) and are available at GitHub: https://github.com/fatimahzachariahali/LAICPMS_protocol_paper_analysis. The code includes: 1. Image masking and phosphorus normalization routines 2. Radial binning and profile extraction 3. Signal-to-background and CV calculations 4. Volumetric reconstruction and integration All scripts required to reproduce the quantitative analyses presented in this study are provided.

## Notes

Composition of embedding media and cryostat sectioning temperatures (Table S1); spherical index and diameter time-course for HT29 and HCT116 spheroids from Day 1 to Day 14 of culture (Figure S1); representative matrix-matched calibration curve for quantitative boron imaging by LA-ICP-MS with 95th percentile confidence intervals (Figure S2); schematic of volumetric reconstruction strategy from 2D LA-ICP-MS sections assuming spherical geometry (Figure S3); estimated volumetric error introduced by the spherical approximation for ellipsoidal spheroids with semiaxes varying up to ± 10% from mean radius (Table S2); radial boron concentration profiles comparing PI-stained and unstained spheroid sections with coefficient of variation analysis (Figure S4); protein expression consistency across seven consecutive 10 μm cryosections for Ki67, GLUT1, and LAT1 in HCT116 and HT29 spheroids (Figure S5); region-wise and radial profiling workflows for correlation of boron signal with immunohistochemical protein expression (Figure S6); representative LA-ICP-MS maps and quantified boron uptake comparing 100 μg mL^–1^ and 1000 μg mL^–1^ BPA incubation concentrations in HCT116 and HT29 spheroids (Figure S7); criteria for interpretation of Pearson correlation coefficients (Table S3) (PDF)

## References

[1] Günther D., Hattendorf B. (2005). Solid sample analysis using laser ablation inductively
coupled plasma mass spectrometry. TrAC Trends
Anal. Chem..

[2] Becker J. S. (2002). Applications
of inductively coupled plasma mass spectrometry and laser ablation
inductively coupled plasma mass spectrometry in materials science. Spectrochim. Acta Part B At. Spectrosc..

[3] Limbeck A., Galler P., Bonta M. (2015). Recent advances in quantitative
LA-ICP-MS analysis: challenges and solutions in the life sciences
and environmental chemistry. Anal. Bioanal.
Chem..

[4] Janovszky P., Kéri A., Palásti D. J. (2023). Quantitative elemental
mapping of biological tissues by laser-induced breakdown spectroscopy
using matrix recognition. Sci. Rep..

[5] Sawicki J., Feldo M., Skalska-Kamińska A., Sowa I. (2025). Modern Bioimaging
Techniques for Elemental Tissue Analysis: Key Parameters, Challenges
and Medical Impact. Molecules.

[6] Schmollinger S., Chen S., Merchant S. S. (2023). Quantitative
elemental imaging in
eukaryotic algae. Metallomics.

[7] Kroslakova I., Günther D. (2007). Elemental fractionation in laser ablation-inductively
coupled plasma-mass spectrometry: evidence for mass load induced matrix
effects in the ICP during ablation of a silicate glass. J. Anal. At. Spectrom..

[8] Becker J. S., Zoriy M. V., Pickhardt C., Palomero-Gallagher N., Zilles K. (2005). Imaging of Copper, Zinc, and Other Elements in Thin
Section of Human Brain Samples (Hippocampus) by Laser Ablation Inductively
Coupled Plasma Mass Spectrometry. Anal. Chem..

[9] Theiner S., Schreiber-Brynzak E., Jakupec M. A. (2016). LA-ICP-MS imaging in
multicellular tumor spheroids – a novel tool in the preclinical
development of metal-based anticancer drugs. Metallomics.

[10] Cruz-Alonso M., Fernandez B., Navarro A. (2019). Laser ablation ICP-MS
for simultaneous quantitative imaging of iron and ferroportin in hippocampus
of human brain tissues with Alzheimer’s disease. Talanta.

[11] Reifschneider O., Schütz C. L., Brochhausen C. (2015). Quantitative bioimaging
of p-boronophenylalanine in thin liver tissue sections as a tool for
treatment planning in boron neutron capture therapy. Anal. Bioanal. Chem..

[12] Konz I., Fernández B., Fernández M. L. (2014). Quantitative bioimaging
of trace elements in the human lens by LA-ICP-MS. Anal. Bioanal. Chem..

[13] Becker J. S., Matusch A., Wu B. (2014). Bioimaging mass spectrometry
of trace
elements – recent advance and applications of LA-ICP-MS: A
review. Anal. Chim. Acta.

[14] Gorman B. L., Torti S. V., Torti F. M., Anderton C. R. (2024). Mass spectrometry
imaging of metals in tissues and cells: methods and biological applications. Biochim. Biophys. Acta Gen. Subj..

[15] Davison C., Beste D., Bailey M., Felipe-Sotelo M. (2023). Expanding
the boundaries of atomic spectroscopy at the single-cell level: critical
review of SP-ICP-MS, LIBS and LA-ICP-MS advances for the elemental
analysis of tissues and single cells. Anal.
Bioanal. Chem..

[16] Willner J., Brunnbauer L., Larisegger S. (2023). A versatile approach
for the preparation of matrix-matched standards for LA-ICP-MS analysis
– Standard addition by the spraying of liquid standards. Talanta.

[17] Miliszkiewicz N., Walas S., Tobiasz A. (2015). Current approaches
to calibration
of LA-ICP-MS analysis. J. Anal. At. Spectrom..

[18] Bonta M., Limbeck A. (2018). Metal analysis in polymers
using tandem LA-ICP-MS/LIBS:
eliminating matrix effects using multivariate calibration. J. Anal. At. Spectrom..

[19] Billimoria K., Menero-Valdes P., Lee W., Shard A., Infante H. G. (2026). Towards
improved workflows for the production and metrological characterization
of LA-ICP-MS calibration standards for quantitative bioimaging. J. Anal. At. Spectrom..

[20] Kuczelinis F., Petersen J. H., Weis P., Bings N. H. (2020). Calibration of LA-ICP-MS
via standard addition using dried picoliter droplets. J. Anal. At. Spectrom..

[21] Sajnóg A., Hanć A., Koczorowski R., Makuch K., Barałkiewicz D. (2018). Usefulness
of laser ablation ICP-MS for analysis of metallic particles released
to oral mucosa after insertion of dental implants. J. Trace Elem. Med. Biol..

[22] Cui Z., He M., Chen B., Hu B. (2023). In-situ elemental quantitative
imaging
in plant leaves by LA-ICP-MS with matrix-matching external calibration. Anal. Chim. Acta.

[23] Wang S., Zhang Z., Miao L., Li Y. (2022). Boron Neutron
Capture
Therapy: Current Status and Challenges. Front.
Oncol..

[24] Das B. C., Nandwana N. K., Das S. (2022). Boron Chemicals in Drug
Discovery and Development: Synthesis and Medicinal Perspective. Molecules.

[25] Verbakel W. F. A. R., Sauerwein W., Hideghety K., Stecher-Rasmussen F. (2003). Boron concentrations
in brain during boron neutron capture therapy: in vivo measurements
from the Phase I trial EORTC 11961 using a gamma-ray telescope. Int. J. Radiat. Oncol..

[26] Prejac J., Skalny A. A., Grabeklis A. R. (2018). Assessing the boron
nutritional status by analyzing its cummulative frequency distribution
in the hair and whole blood. J. Trace Elem.
Med. Biol..

[27] Pollmann D., Broekaert J. A. C., Leis F., Tschöpel P., Tölg G. (1993). Determination of boron in biological tissues by inductively
coupled plasma optical emission spectrometry (ICP-OES). Fresenius J. Anal. Chem..

[28] Kronenberg K., Hoffmann E., Hiddeßen L. (2024). P-based referencing
for correcting tissue artifacts in laser ablation-inductively coupled
plasma-mass spectrometry imaging of cancer samples. Metallomics.

[29] Vonderach T., Gundlach-Graham A., Günther D. (2024). Determination of carbon in microplastics
and single cells by total consumption microdroplet ICP-TOFMS. Anal. Bioanal. Chem..

[30] Kritmetapak K., Kumar R. (2021). Phosphate as a Signaling Molecule. Calcif.
Tissue Int..

[34] Rao K. R. P., Ray V. H., Patel A. R. (1983). Serum ferritin and sequestered stores
of body iron. Am. J. Clin. Pathol..

[35] Maret W., Krezel A. (2007). Cellular Zinc and Redox
Buffering Capacity of Metallothionein/Thionein
in Health and Disease. Mol. Med..

[36] Rosenzweig A. C. (2001). Copper
delivery by metallochaperone proteins. Acc.
Chem. Res..

[37] Lister, A. S. Validation of HPLC Methods in Pharmaceutical Analysis. In Sep. Sci. Technol.; Academic Press, 2005; Vol. 6, pp 191–217.

[38] Hansen A. W., Venkatachalam K. V. (2023). Sulfur-Element containing metabolic pathways in human
health and crosstalk with the microbiome. Biochem.
Biophys. Rep..

[39] Römpp A., Spengler B. (2013). Mass spectrometry imaging with high resolution in mass
and space. Histochem. Cell Biol..

[31] Schweikert A., Theiner S., Wernitznig D. (2022). Micro-droplet-based
calibration for quantitative elemental bioimaging by LA-ICPMS. Anal. Bioanal. Chem..

[32] Šala M., Šelih V. S., van Elteren J. T. (2017). Gelatin
gels as multi-element calibration
standards in LA-ICP-MS bioimaging: fabrication of homogeneous standards
and microhomogeneity testing. Analyst.

[33] Thoröe-Boveleth S., Becker R., Bertram J. (2025). Line-dropped gelatin
multi-element calibration standards in LA-ICP-MS: a statistically
verifying comparison with cryosectioned homogenized lung and liver
as matrix-matched calibration standards and as corresponding reference
materials. Anal. Sci..

[40] Akoglu H. (2018). User’s
guide to correlation coefficients. Turk. J.
Emerg. Med..

[41] Buchholz R., Krossa S., Andersen M. K. (2022). A simple preparation
protocol for shipping and storage of tissue sections for laser ablation-inductively
coupled plasma-mass spectrometry imaging. Metallomics.

[42] Frick D. A., Giesen C., Hemmerle T., Bodenmiller B., Günther D. (2015). An internal standardisation strategy for quantitative
immunoassay tissue imaging using laser ablation inductively coupled
plasma mass spectrometry. J. Anal. At. Spectrom..

[43] Austin C., Fryer F., Lear J. (2011). Factors affecting internal
standard selection for quantitative elemental bio-imaging of soft
tissues by LA-ICP-MS. J. Anal. At. Spectrom..

[44] Petrelli M., Laeger K., Perugini D. (2016). High spatial
resolution trace element
determination of geological samples by laser ablation quadrupole plasma
mass spectrometry: implications for glass analysis in volcanic products. Geosci. J..

[45] Austin C., Hare D., Rawling T., McDonagh A. M., Doble P. (2010). Quantification
method for elemental bio-imaging by LA-ICP-MS using metal spiked PMMA
films. J. Anal. At. Spectrom..

[46] Hsiao I.-L., Bierkandt F. S., Reichardt P. (2016). Quantification and visualization
of cellular uptake of TiO2 and Ag nanoparticles: comparison of different
ICP-MS techniques. J. Nanobiotechnol..

[47] Egger A. E., Theiner S., Kornauth C. (2014). Quantitative
bioimaging
by LA-ICP-MS: a methodological study on the distribution of Pt and
Ru in viscera originating from cisplatin- and KP1339-treated mice†. Metallomics.

[48] Cernatič F., Brunnbauer L., Mervič K. (2025). Signal Perception in
Two-Dimensional Mapping Techniques: Just Noticeable Difference as
a Visual Limit of Detection. Anal. Chem..

[49] Quílez C., González-Rico J., López-Donaire M. L. (2026). Absolute
Quantification and Spatial Mapping of Hyaluronic Acid in Histological
Tissue Sections. ACS Meas. Sci. Au.

[50] Libard S., Cerjan D., Alafuzoff I. (2019). Characteristics
of the tissue section
that influence the staining outcome in immunohistochemistry. Histochem. Cell Biol..

[51] Jantarat T., Doungchawee J., Zhang X., Rotello V. M., Vachet R. W. (2025). Image Fusion
for Improving the Spatial Resolution of LA-ICP-MS Imaging. Anal. Chem..

[52] Michel T., Fehrenbach J., Lobjois V. (2018). Mathematical
modeling
of the proliferation gradient in multicellular tumor spheroids. J. Theor. Biol..

[53] Mertzanidou T., Hipwell J. H., Reis S. (2017). 3D volume
reconstruction
from serial breast specimen radiographs for mapping between histology
and 3D whole specimen imaging. Med. Phys..

[54] van
Elteren J. T., Metarapi D., Mervič K., Šala M. (2023). Exploring the Benefits of Ablation Grid Adaptation
in 2D/3D Laser Ablation Inductively Coupled Plasma Mass Spectrometry
Mapping through Geometrical Modeling. Anal.
Chem..

