# Supplementary material for: Quantitative LA-ICP-MS Imaging of Elemental Distributions with Protein Correlation in 3D Tumor Models
Source: Anal Chem. 2026 Jul 30;98(31):23164–78. doi: 10.1021/acs.analchem.6c03012 (PMC13470975; doi:10.1021/acs.analchem.6c03012)
Supplement: Supplementary file 1 [file ac6c03012_si_001.pdf]

Supplementary Information

Quantitative LA-ICP-MS imaging of elemental distributions with protein correlation in 3D tumour models

Fatimah Zachariah Ali[1,2]*, Alexander P. Morrell[3], Piotr Robert Golda[3], Norfazlina Mohd Nawi[4], Premkamon Chaipanichkul[1,5], John M. McArthur[6], Pascal F. Durrenberger[7], Huda Alnufaei[1], Gary Royle[8], Kate Ricketts[1]*

1. Division of Surgery and Interventional Science, Faculty of Medicine, University College London (UCL), 43-45 Foley St, London, W1W 7TY, United Kingdom (UK)
2. Department of Biomedical Imaging, Faculty of Medicine, Universiti Malaya, South Tower, Universiti Malaya Medical Centre (UMMC), Jalan Universiti, Lembah Pantai 59100 Kuala Lumpur, Malaysia
3. London Metallomics Facility, King's College London (KCL), Franklin Wilkins Building, Stamford Street, London, SE1 9NQ, UK
4. Institute for Biological Science, Faculty of Science, Universiti Malaya, Lembah Pantai, Wilayah Persekutuan, 50603 Kuala Lumpur, Malaysia
5. Division of Radiation Oncology, Department of Radiology, Faculty of Medicine, Khon Kaen University, 123 Mittraphap Highway, Nai Mueang Sub-District, Mueang Khon Kaen District, Khon Kaen 40002, Thailand
6. Department of Earth Sciences, UCL, 5 Gower Pl, London, WC1E 6BS, UK
7. Respiratory Medicine, Faculty of Medicine, UCL, 5 University Street, London, WC1E 6JF, UK
8. Department of Medical Physics & Biomedical Engineering, UCL, Malet Place Engineering Building, Torrington Place, London, WC1E 7JE, UK

***Corresponding authors;** Fatimah Zachariah Ali (fatimah.ali.16@ucl.ac.uk), Kate Ricketts (k.ricketts@ucl.ac.uk)

Table of Contents

Table S1: Composition of embedding media used under different conditions 3

Figure S1: Spheroids' spherical index and diameter time-course 4

Figure S2: Quantitative imaging via matrix-matched calibration standards (calibration curve).. 5

Figure S3: Volumetric reconstruction of spheroids content 6

Table S2: Ellipsoid volume error estimation 7

Figure S4: PI staining vs no PI staining comparison 8

Figure S5: Consecutive section protein validation 9

Figure S6: Proteins correlation method analysis 11

Figure S7: 100 µg/mL vs 1000 µg/mL BPA comparison 12

Table S3: Pearson coefficient correlation interpretation 13

Table S1: Composition of embedding media used under different conditions

*Table S1: **Composition of embedding media used under different conditions.** All mixtures were continuously stirred on a magnetic hot plate at 70 °C until fully dissolved and transparent. The prepared media were subsequently stored at 4 °C overnight prior to use.*

**Embedding media**	**Mass needed for 100mL end volume**		**Sample temperature during sectioning (°C)**
	Mili-Q water (mL)	*Powder (g)*	
**OCT**	-	-	-20
**5% Gelatine**	100	5 (Gelatine)	-7
**2% CMC + 5 % Gelatine**	100	5 (Gelatine) + 2 (CMC)	-5
**2% CMC**	100	2 (CMC)	-5

Figure S1: Spheroids' spherical index and diameter time-course

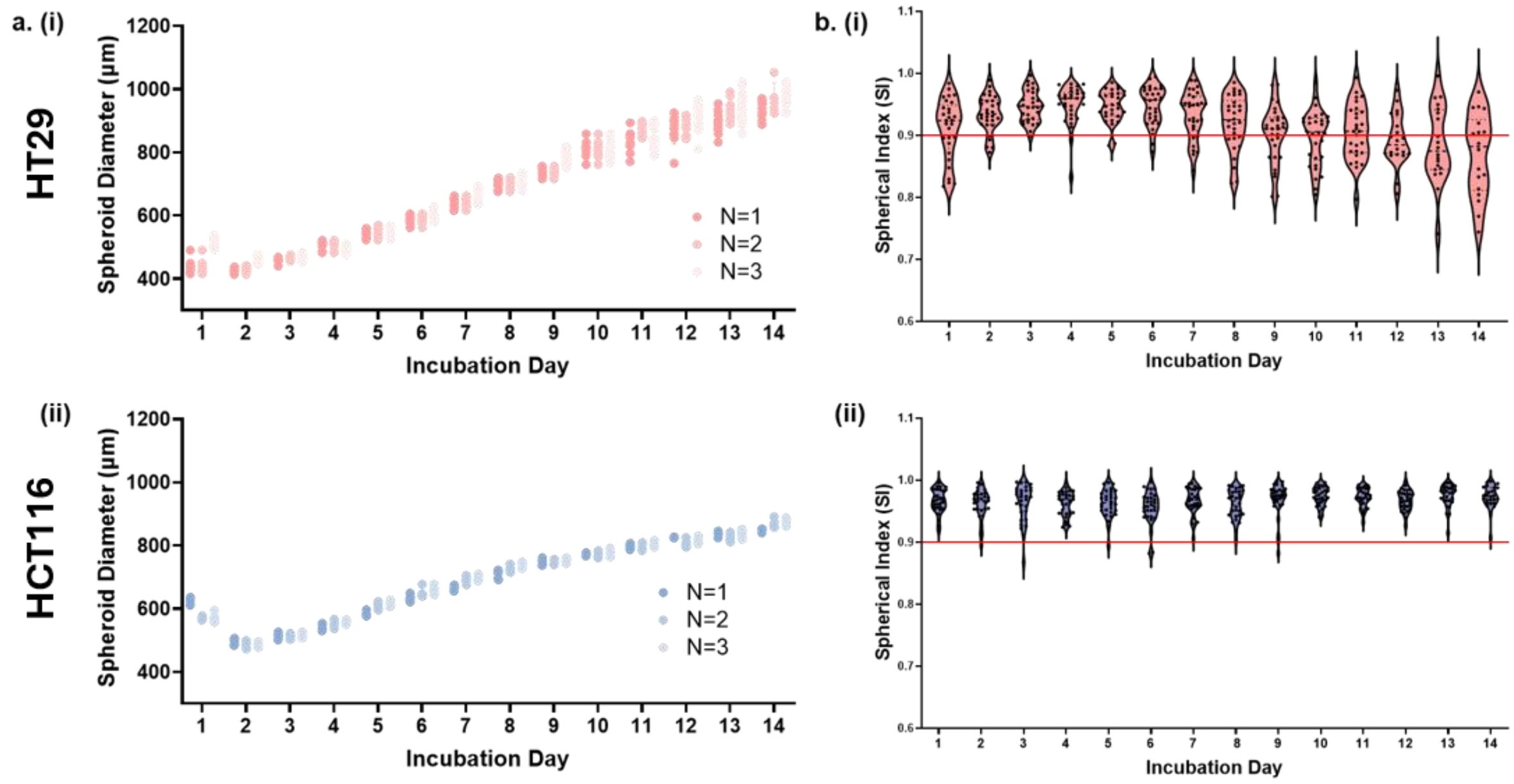

Figure S1: ***Spherical index of HT29 and HCT116 multicellular tumour spheroids from Day 1 to Day 14 of culture.*** *Spherical index (SI) was calculated from brightfield images acquired daily using the formula SI = 4πA/P², where A is the projected area and P is the perimeter of the spheroid cross-section. Values approaching 1.0 indicate perfect sphericity. Both cell lines consistently achieved SI > 0.9 by Day 7, the timepoint selected for all experiments in this study. Data are presented as mean ± SD (n = 10 spheroids per timepoint). The dashed line indicates SI = 0.9.*

Figure S2: Quantitative imaging via matrix-matched calibration standards (calibration curve)

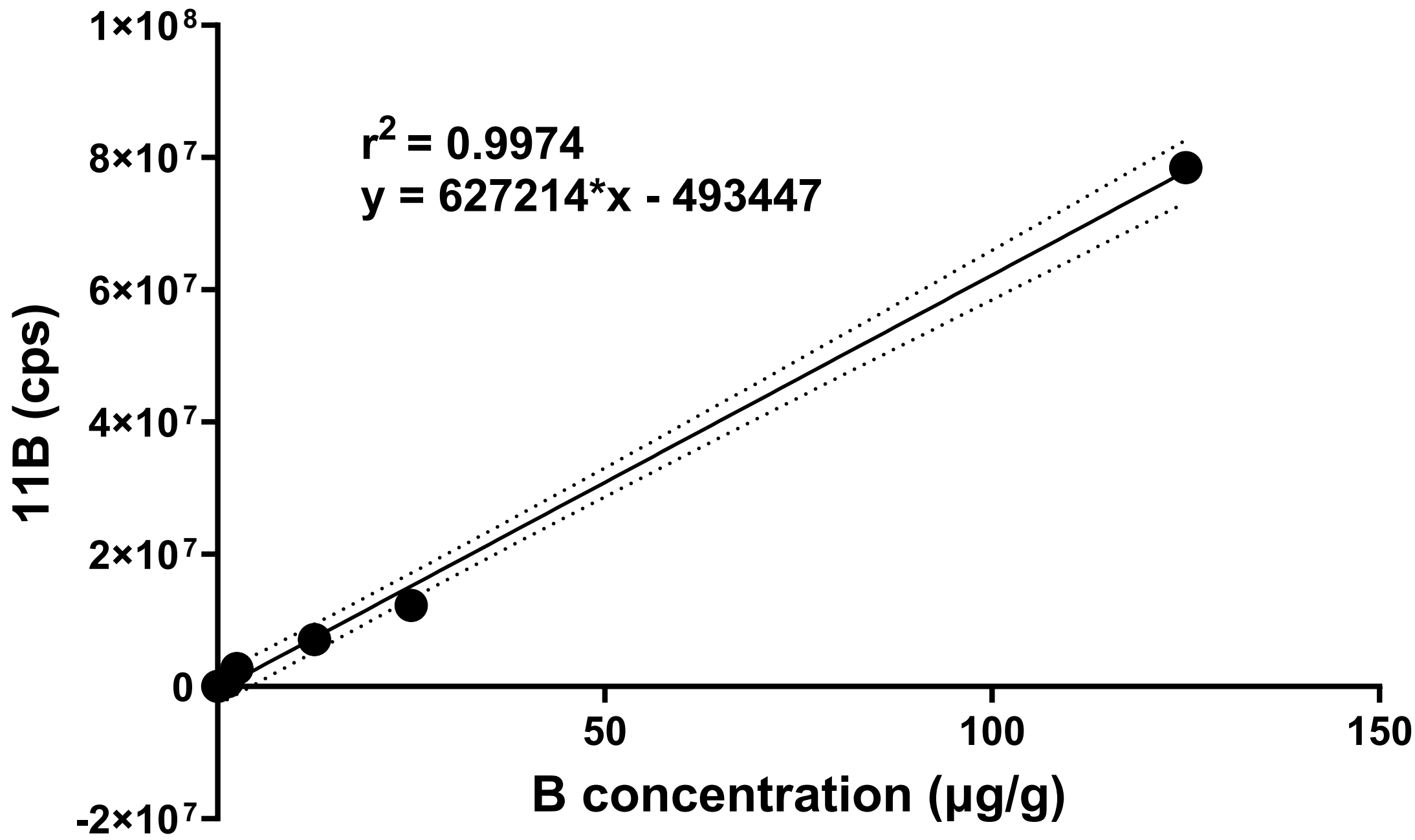

*Figure S2**: Matrix-matched calibration curve for quantitative boron imaging by LA-ICP-MS.** Representative calibrated boron concentration obtained from B-spiked fish gelatine of known concentrations for the LA-ICP-MS run. Linear calibration curve generated from boron-spiked fish gelatine standards (0.5–50 mg L^{-1}) analysed within the same run as spheroid sections. Signal intensity (counts per second) is plotted against known boron concentration. The relationship was highly linear across the working range ($r^2 > 0.99$) and reproducible across three independent runs, supporting conversion of elemental signal intensity to absolute concentration (µg g^{-1}) for quantitative imaging. The dotted lines show the 95th percentile.*

Figure S3: Volumetric reconstruction of spheroids content

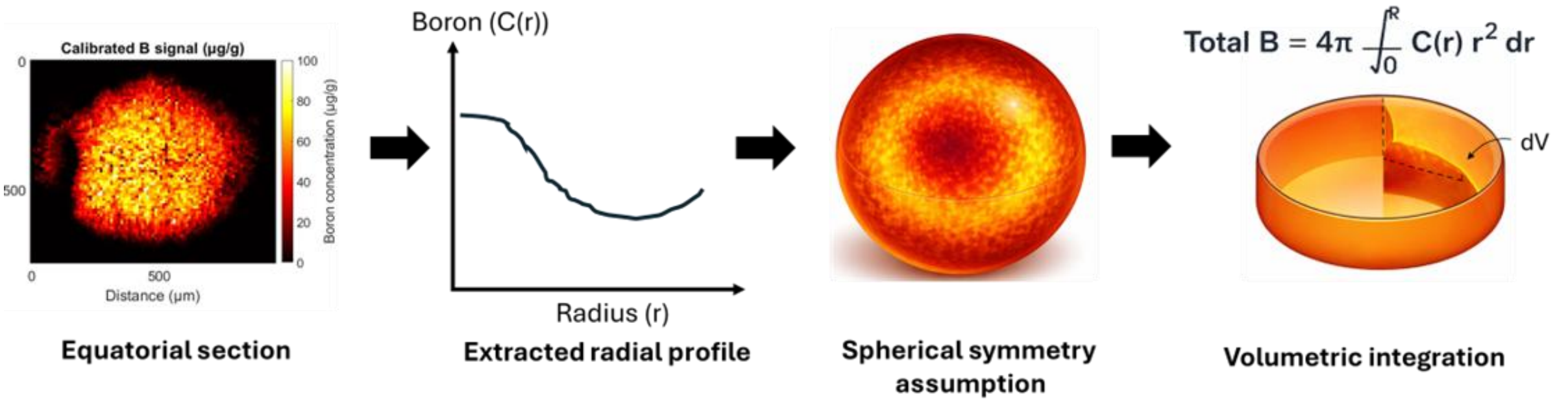

Fig. S3: ***Schematic of volumetric reconstruction strategy.*** *The analysed 30-µm section is assumed to represent the equatorial plane of a spherical spheroid; the radially resolved boron distribution is propagated along the z-axis to generate a three-dimensional concentration map. Pixel-wise calibrated concentrations are converted to voxels under spherical symmetry assumptions and integrated to compute total boron content per spheroid.*

Table S2: Ellipsoid volume error estimation

Table S2: ***Estimated volumetric error introduced by the spherical approximation for ellipsoidal spheroids.*** *Legend: Volume error was calculated by comparing the volume of an ellipsoid ($V = 4/3\pi abc$) to that of a sphere of equivalent mean radius ($V = 4/3\pi r^3$), where $r = 1$ (normalised). Semi-axes a, b, and c are expressed as multiples of r. A spherical index (SI) > 0.9, as observed for all spheroids used in this study at Day 7, corresponds to axis deviations of ≤10% from the mean radius. Positive values indicate volume overestimation; negative values indicate underestimation. Volume error (%) = [(V_ellipsoid − V_sphere) / V_sphere] × 100.*

Shape / Axis Configuration	a	b	c	Volume Error (%)
**Perfect sphere**	1.00r	1.00r	1.00r	0 (reference)
**Mildly oblate (a=b=1.05r, c=0.95r)**	1.05r	1.05r	0.90r	-0.8
**Mildly prolate (a=b=0.95r, c=1.05r)**	0.95r	0.95r	1.10r	-0.7
**Oblate (a=b=1.10r, c=0.90r)**	1.10r	1.10r	0.90r	+8.9
**Prolate (a=b=0.90r, c=1.10r)**	0.90r	0.90r	1.10r	-10.9
**Asymmetric (a=1.10r, b=1.00r, c=0.90r)**	1.10r	1.00r	0.90r	-1.0

Figure S4: PI staining vs no PI staining comparison

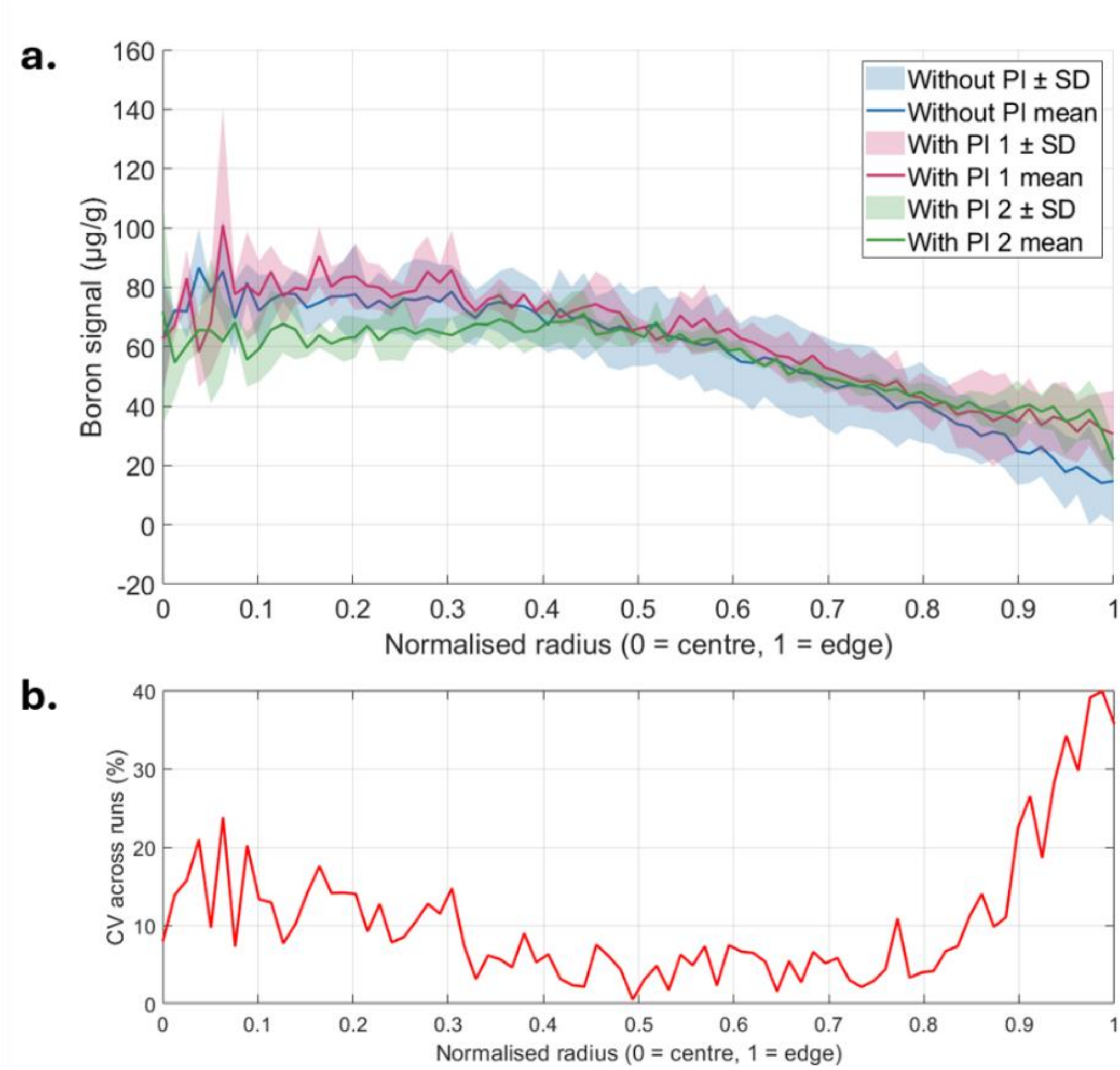

Figure S4: ***Effect of propidium iodide staining on spatial boron distribution in tumour spheroid sections.*** *(a) Radial boron concentration profiles (µg g^{-1}) extracted from spheroid sections processed without PI staining (blue) and two independent PI-stained runs (pink, green), plotted as mean ± SD as a function of normalised radius (0 = spheroid centre, 1 = spheroid edge). All three profiles follow the same spatial distribution pattern, with overlapping SD bands across the majority of the spheroid radius, confirming that PI incubation does not introduce a detectable artefact in boron spatial distribution. Pairwise Pearson correlations between all conditions were strong and statistically significant ($r > 0.928$, $p < 0.0001$). (b) Coefficient of variation (CV, %) across the three conditions as a function of normalised radius. CV was below 20% across most of the spheroid (normalised radius 0.1–0.85), with higher variability observed at the periphery (normalised radius > 0.85), consistent with reduced signal intensity and increased noise contribution at the spheroid boundary. These data confirm that the PI-based dead-cell mask can be applied to LA-ICP-MS quantification without confounding the measured elemental signal.*

Figure S5: Consecutive section protein validation

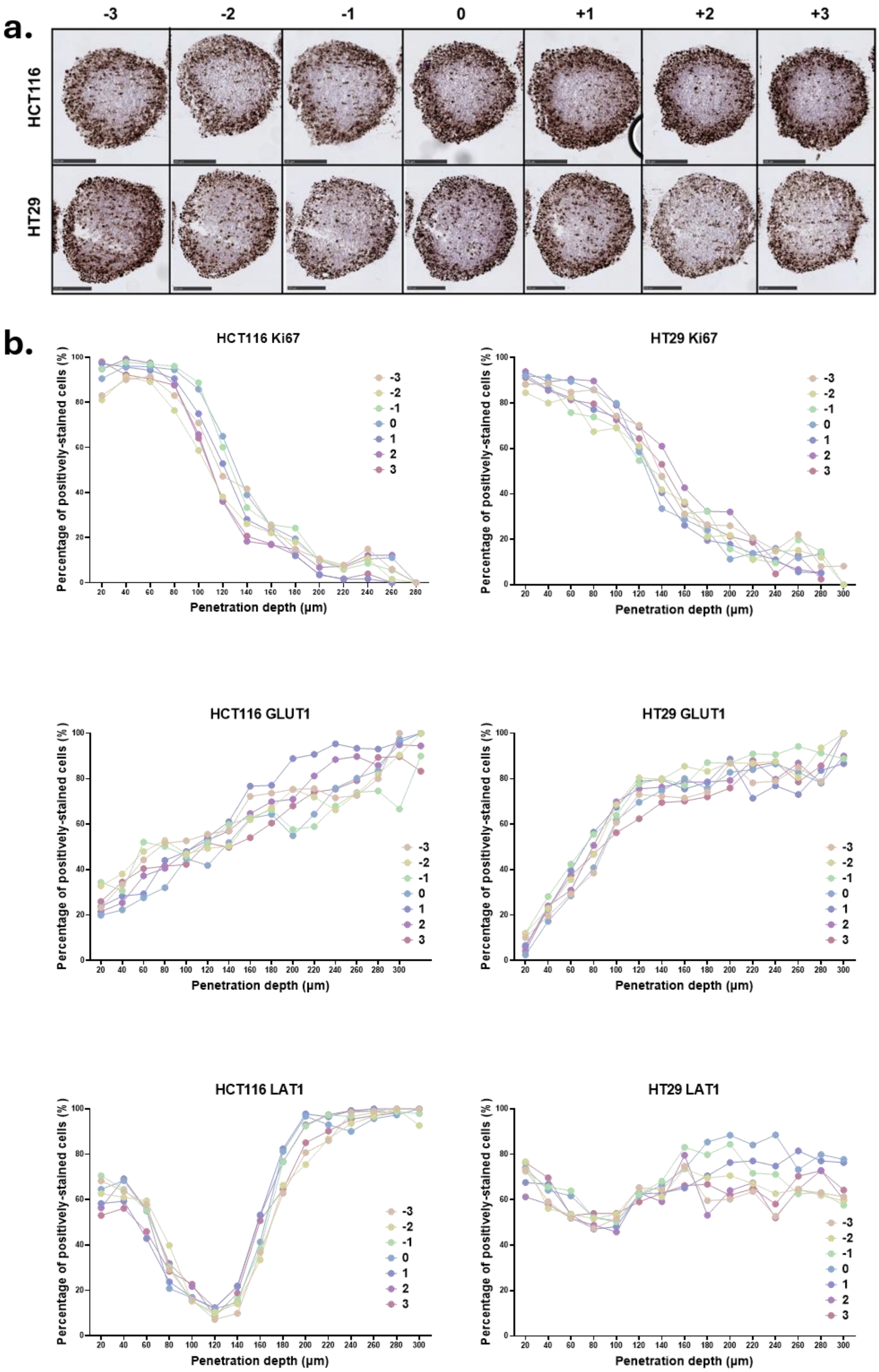

Figure S5: ***Protein expression consistency across seven consecutive 10 µm cryosections of multicellular tumour spheroids.*** *(a) Representative brightfield images of Ki67-stained sections from HCT116 (top row) and*

HT29 (bottom row) spheroids across seven consecutive 10 μm sections, numbered relative to the central section (0). Section 0 corresponds to the section used for LA-ICP-MS analysis; sections −3 to +3 span a total depth of 60 μm on either side. The spatial pattern of Ki67 staining is visibly consistent across all sections in both cell lines. Scale bar = 200 μm. (b) Radial penetration depth profiles showing the percentage of positively stained cells as a function of penetration depth from the spheroid periphery, for Ki67, GLUT1, and LAT1 in HCT116 (left column) and HT29 (right column) spheroids. Marker-dependent differences in section-to-section variability were observed. Ki67 and GLUT1 showed tight agreement across all seven sections in both cell lines (CV < 20%). LAT1 in HT29 spheroids exhibited greater variability (CV 20–40%), likely reflecting its lower and more spatially heterogeneous expression pattern in this cell line, which amplifies relative variation for equivalent absolute differences between sections. Based on this observation, we recommend that for markers exhibiting high spatial heterogeneity or low absolute expression — such as LAT1 in HT29 — IHC staining should be performed on the section immediately adjacent (within one section, i.e., 10–30 μm) to the LA-ICP-MS section to minimise depth-dependent discordance.

Figure S6: Proteins correlation method analysis

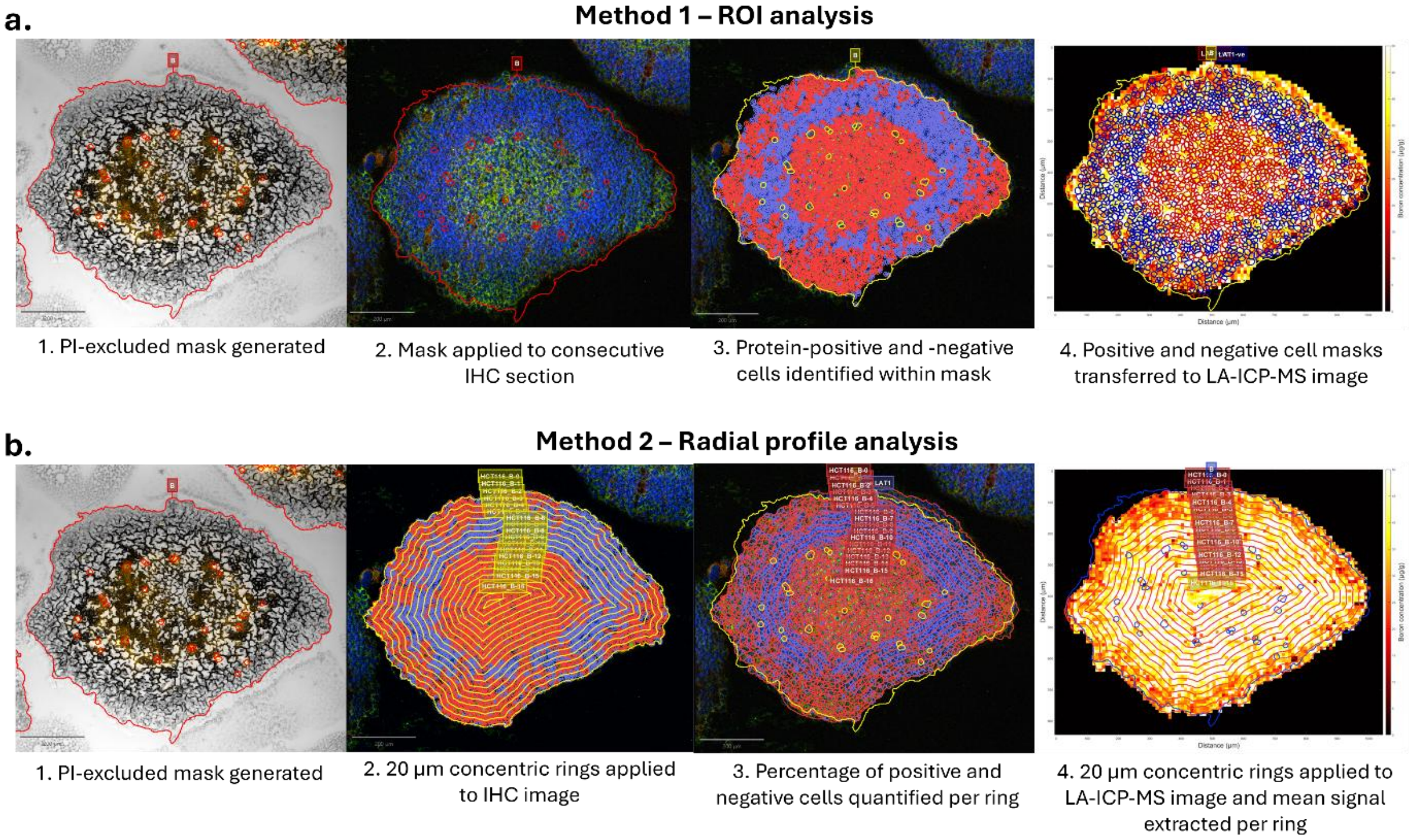

Figure S6: ***Region-wise and radial correlation of boron signal with immunohistochemical protein expression in consecutive spheroid sections****. (a) Region-of-interest (ROI) correlation workflow illustrating PI exclusion, segmentation of protein positive and negative regions, and extraction of corresponding boron signal. Differences in B signal between protein-positive and protein-negative regions were assessed using an unpaired two-tailed t-test. (b) Radial profile correlation workflow comparing mean normalised boron and IHC intensities across concentric rings (20 μm width) from spheroid centre to periphery.*

Figure S7: 100 µg/mL vs 1000 µg/mL BPA comparison

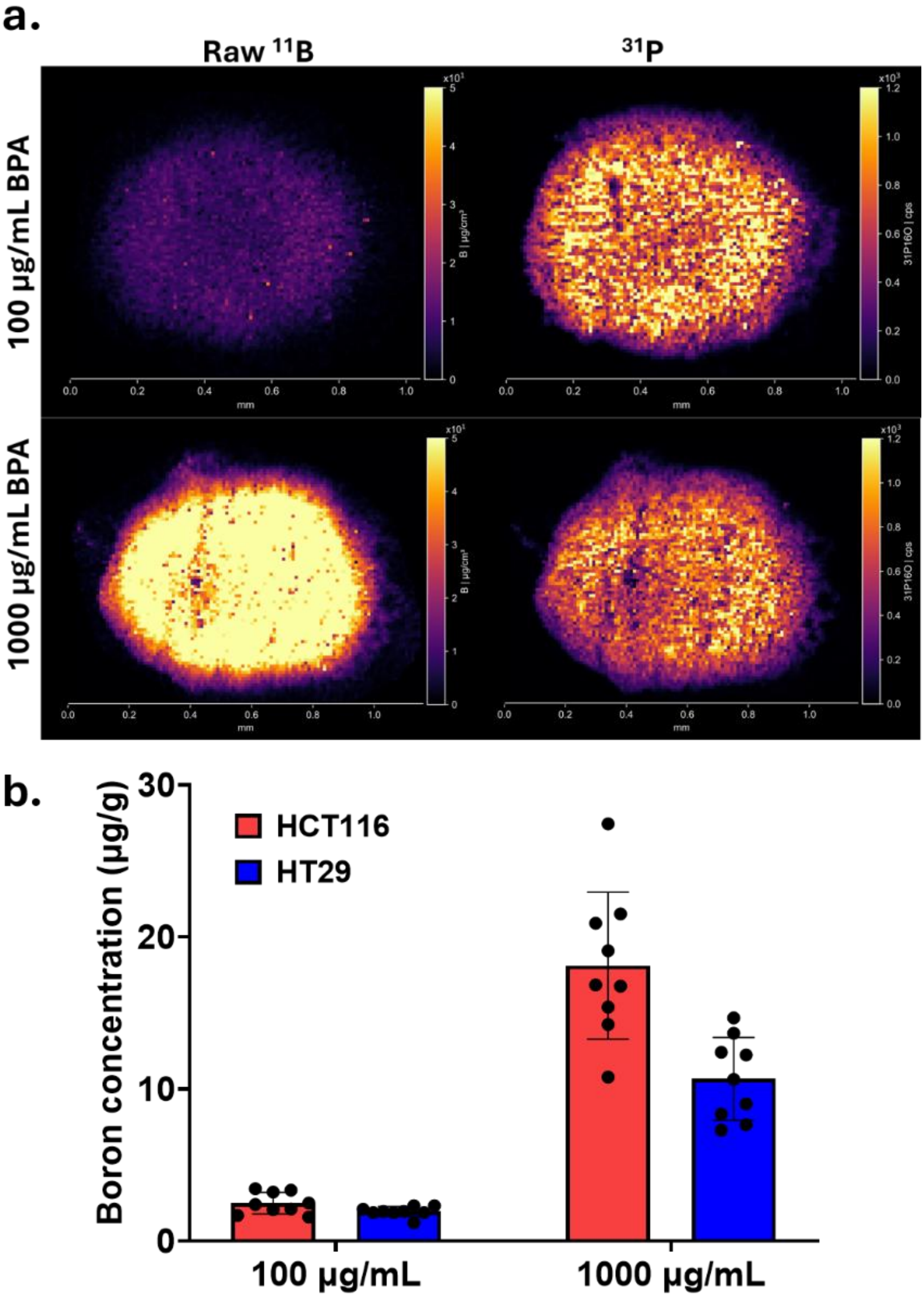

Figure S7: ***Effect of BPA incubation concentration on spheroid boron uptake and spatial distribution.*** *(a) Representative raw ^{11}B and ^{31}P LA-ICP-MS maps of HCT116 spheroids incubated with 100 µg/mL or 1000 µg/mL BPA, showing spatial boron distribution and spheroid morphology respectively. (b) Mean boron concentration (µg g^{-1}) quantified by LA-ICP-MS in HCT116 and HT29 spheroids at both incubation concentrations (n = 3 per condition, 3 spheroids per replicate; mean ± SD). At 100 µg/mL, tissue boron levels were insufficient to reach the therapeutically relevant range for BNCT (approximately 20–50 µg g^{-1}), whereas 1000 µg/mL produced uptake levels approaching or within this range in a cell-line-dependent manner. Both concentrations exhibited comparable spatial distribution patterns, confirming that the peripheral signal observed at 1000 µg/mL is a property of the embedding protocol rather than a concentration-dependent artefact.*

Table S3: Pearson coefficient correlation interpretation

*Table S3: **Criteria for interpretation of Pearson correlation coefficients used in this study.** Correlation strength was defined according to Akoglu et. al, 2018. These thresholds were applied consistently across all Pearson r values reported in this study.*

Correlation coefficient	Degree of correlation
0.80 to 1.00 (-0.80 to -1.00)	Very high
0.6 to 0.8 (-0.6 to -0.8)	High
0.4 to 0.6 (-0.4 to -0.6)	Medium
0.2 to 0.4 (-0.2 to -0.4)	Low
0.00 to 0.20 (0.00 to – 0.20)	Very low
